# Effects of combined aerobic and resistance training on cardiometabolic risk factors in overweight/obese adolescents: a systematic review and meta-analysis

**DOI:** 10.3389/fpubh.2026.1891252

**Published:** 2026-07-06

**Authors:** Junjian Wang, Jiayi Yao, Wenjia Chen

**Affiliations:** 1 School of Wushu, Shandong Sport University, Jinan, China; 2 School of Physical Education, China University of Mining and Technology, Xuzhou, China

**Keywords:** adiponectin, adolescents, blood glucose, blood pressure, combined training, meta-analysis, obesity

## Abstract

**Background and objective:**

Implementing scientific exercise interventions during childhood and adolescence holds long-term value for vascular protection. This study aimed to systematically evaluate the actual effects of combined aerobic and resistance training (CART) on glucose metabolism parameters, lipid profiles, and blood pressure dynamics in adolescents with overweight or obesity.

**Methods:**

Electronic databases including PubMed, Web of Science, Embase, The Cochrane Library, and Scopus were searched from inception up to May 13, 2026, to collect randomized controlled trials (RCTs) investigating CART interventions in adolescents with overweight or obesity. Data analysis was performed using R software. Mean difference (MD) and 95% confidence intervals (CI) were uniformly employed as effect measures, with change-from-baseline values entered into the models. Hierarchical subgroup analyses based on control group type were conducted utilizing the Hartung-Knapp-Sidik-Jonkman (HKSJ) random-effects model, and the quality of evidence was assessed using the Grading of Recommendations Assessment, Development and Evaluation (GRADE) approach.

**Results:**

A total of 12 RCTs were included. Regarding glucose metabolism, fasting insulin showed a significant reduction in the CART vs. non-exercise control (CON) subgroup (MD = −17.90 μU/mL, 95% CI: −26.04 to −9.76, *P* < 0.0001), and also demonstrated a notable tendency toward improvement in the CART vs. aerobic exercise (AE) subgroup (MD = −2.70 μU/mL, 95% CI: −4.61 to −0.79, *P* = 0.01), with a statistically significant difference observed between the two subgroups (*P* = 0.0004). The homeostatic model assessment of insulin resistance (HOMA-IR) demonstrated a tendency toward improvement in the CART vs. AE subgroup (MD = −0.57, 95% CI: −1.01 to −0.12, *P* = 0.01). Adiponectin demonstrated a notable tendency toward elevation in the CART vs. AE subgroup (MD = 2.37 μg/mL, 95% CI: 1.53 to 3.21, *P* < 0.0001). Fasting glucose showed no notable change in either subgroup and exhibited extremely high heterogeneity, making it inappropriate to draw substantial conclusions. Regarding blood pressure, systolic blood pressure (SBP) and diastolic blood pressure (DBP) both demonstrated a notable tendency toward reduction in the CART vs. CON subgroup (SBP: MD = −3.51 mmHg, 95% CI: −6.23 to −0.80, *P* = 0.01; DBP: MD = −3.72 mmHg, 95% CI: −6.69 to −0.75, *P* = 0.01), whereas neither showed notable change in the CART vs. AE subgroup. Total cholesterol and triglycerides showed no notable change in any subgroup. The GRADE evidence quality assessment showed that fasting insulin, SBP, and DBP in the CART vs. CON subgroup, as well as fasting insulin and HOMA-IR in the CART vs. AE subgroup, were of moderate quality, while the remaining indicators were of low or very low quality.

**Conclusion:**

Current evidence indicates that CART demonstrates statistically significant improvements in fasting insulin, SBP, DBP, and adiponectin levels in adolescents with overweight or obesity; however, due to limitations including high variability in intervention protocols, absence of blinding, and regional concentration of included studies, the overall quality of evidence is low, and the actual clinical meaningfulness still requires cautious interpretation. All conclusions must be interpreted based on the classification of control group activity status. Future research urgently needs to adopt standardized combined training protocols, establish consistent comparator definitions, and conduct large-sample RCTs with longer follow-up periods for confirmation.

## 1 Introduction

Cardiovascular disease (CVD) is the leading cause of death worldwide, with the accumulation of its risk factors beginning as early as childhood and adolescence (1, 2). In recent years, with the increasing prevalence of sedentary behavior and unhealthy dietary patterns, the rates of overweight and obesity among adolescents have continued to rise, and the age of onset of cardiometabolic risk factors such as hypertension, dyslipidemia, and insulin resistance has advanced significantly (3–5). A large body of longitudinal evidence confirms that cardiometabolic health during adolescence is closely associated with CVD risk in adulthood, and early intervention in this population holds important public health significance (6, 7).

Among non-pharmacological intervention strategies, exercise training is widely recommended for its high safety and accessibility (8). Combined aerobic and resistance training (CART) has attracted increasing attention in recent years due to its ability to simultaneously target multiple cardiometabolic risk factors (9). Existing research suggests that CART may be superior to single-modality exercise in improving body composition, lipid profiles, and insulin sensitivity; however, the available evidence remains insufficient, particularly given the limited number of high-quality randomized controlled trials conducted in adolescents with overweight or obesity (10, 11).

Despite the growing number of related systematic reviews and meta-analyses, the existing evidence is still subject to two critical methodological bottlenecks that have prevented a full elucidation of the true benefits of the intervention. First, most reviews tend to conflate structurally distinct comparator types, frequently merging inactive non-exercise controls (CON) and active aerobic exercise controls (AE) into a highly heterogeneous matrix. This conceptual conflation inherently dilutes the true physiological incremental value of CART, and certain important outcome indicators such as fasting insulin and HOMA-IR have not yet been subjected to in-depth quantitative analysis due to the scarcity of prior literature or the absence of rigorous parallel comparisons (12). Second, prior meta-analyses in this field have predominantly relied on the traditional DerSimonian-Laird random-effects model, which, when applied to the small-sample, small-cohort studies characteristic of pediatric research, is highly prone to underestimating between-study heterogeneity and generating an elevated false-positive rate, thereby failing to provide high-certainty direct evidence to guide exercise prescription selection in clinical practice. This has made the conduct of a new systematic review one that employs a more robust model to hierarchically isolate evidence particularly urgent (13, 14). Accordingly, the core evidence gap this study seeks to address is precisely focused on the conceptual conflation in “comparator condition separation” present in prior syntheses, as well as the methodological limitations arising from reliance on “traditional statistical models” in the context of small-sample cohorts.

Given that a purely narrative review cannot precisely quantify the net effects of different exercise modalities, nor can it quantitatively disentangle the heterogeneity conflicts arising from comparator conflation in prior syntheses, this study aims to leverage the core statistical corrective and adjudicative value of a higher-level quantitative meta-analysis. To this end, this study systematically searched and included randomized controlled trials to quantitatively evaluate the precise effects of CART on fasting glucose, fasting insulin, homeostatic model assessment of insulin resistance (HOMA-IR), adiponectin, total cholesterol, triglycerides, systolic blood pressure (SBP), and diastolic blood pressure (DBP) in adolescents affected by overweight or obesity. Through a rigorous dual-tier hierarchical subgroup framework, this study separately quantifies and compares the effects of CART relative to non-exercise control (CON) and aerobic exercise-only (AE) groups, as well as the between-subgroup differences. Furthermore, the more robust Hartung-Knapp-Sidik-Jonkman (HKSJ) random-effects adjustment model is introduced with the aim of resolving long-standing efficacy controversies in the field and providing higher-certainty evidence-based guidance for the formulation of exercise prescriptions targeting cardiometabolic risk in adolescents.

## 2 Materials and methods

This study protocol has been registered in PROSPERO (Registration No.: CRD420261394736), with clearly defined research objectives, inclusion and exclusion criteria, intervention and comparator conditions, and outcome indicators. The study was conducted in strict accordance with the pre-registered protocol without major deviations, and was implemented and reported following the Preferred Reporting Items for Systematic Reviews and Meta-Analyses (PRISMA 2020) checklist (15).

### 2.1 Search strategy

This study systematically searched PubMed (via NCBI), Web of Science Core Collection, Scopus, The Cochrane Library, and Embase (via Elsevier) databases on May 13, 2026, covering the period from database inception to that date. No restrictions were placed on the year of publication, and the search language was limited to English. The search strategy was developed based on the PICOS framework, combining controlled vocabulary (e.g., MeSH, Emtree) with free-text terms, and was adapted to the specific characteristics of each database (e.g., TIAB field restrictions, TS full-term matching, and proximity operators). Core terms encompassed “children and adolescents (Child/Adolescent/Youth),” “combined or concurrent training (Combined/Concurrent training),” and “cardiometabolic indicators including arterial stiffness, blood pressure, insulin resistance, and lipids (Arterial stiffness/BP/Insulin resistance/Lipids),” with RCT filters applied for precise screening. In addition, reference lists of included studies were manually tracked and attempts were made to contact original authors to obtain missing data. Gray literature and preprint databases were not searched. The complete search strategies are provided in Supplementary File 1.

### 2.2 Exclusion and inclusion criteria

Inclusion criteria were developed based on the PICOS framework: 1) Population (P): adolescents with overweight or obesity, with an age cut-off of 10–19 years (consistent with the WHO age definition of adolescents); 2) Intervention (I): the experimental group received a combined training program incorporating both aerobic and resistance components (CART); 3) Comparator conditions (C): two clearly defined comparator frameworks were included, namely non-exercise control (CON, including no exercise, maintenance of routine lifestyle, or receipt of usual care) or active single-modality exercise control (specifically aerobic exercise only, AE); 4) Outcomes (O): at least one cardiovascular/metabolic outcome indicator must be reported, including blood pressure [systolic blood pressure (SBP), diastolic blood pressure (DBP)] or subclinical atherosclerosis markers [pulse wave velocity (PWV), carotid intima-media thickness (cIMT), or flow-mediated dilation (FMD)]; 5) Study design (S): RCTs. Studies were excluded if they were non-randomized controlled trials (e.g., animal experiments, case reports, and crossover studies), involved participants with severe systemic diseases, did not include combined training as the intervention, did not report cardiovascular outcome data or had missing data that could not be obtained, or were duplicate publications.

### 2.3 Data collection process and data items

Data extraction was performed independently by two researchers and recorded with reference to the Cochrane Handbook guidelines. Based on the summary table of this study, extracted information included: ① basic study information (author, year of publication); ② participant characteristics (sample size, age, sex, health status/degree of obesity); ③ intervention protocol details (intervention duration, training frequency, specific duration and intensity of aerobic and resistance training components, and control group design); ④ cardiovascular outcome indicators, including SBP, DBP, or subclinical atherosclerosis markers (e.g., PWV, cIMT, FMD). Any discrepancies during the extraction process were resolved through discussion or by consulting a third independent researcher. For studies that reported only pre- and post-intervention independent standard deviations without providing standard deviations for change scores, imputation was performed using the formula recommended in the Cochrane Handbook, with the assumed correlation coefficient (*r*) between pre- and post-intervention values uniformly set at 0.5 (16). In cases of missing or unclear data, original authors were contacted via email for supplementary information.

### 2.4 Risk of bias

The risk of bias of included studies was independently assessed by two researchers using the Cochrane Risk of Bias tool (RoB 2.0) (17). Assessment was conducted across five core domains applicable to RCTs: ① bias arising from the randomization process; ② bias due to deviations from the intended intervention; ③ bias due to missing outcome data; ④ bias in the measurement of outcomes; ⑤ bias in the selection of reported results. During the replication assessment process, the risk level for each domain was rated as “low risk,” “some concerns,” or “high risk” according to the RoB 2.0 signaling questions. The overall risk of bias for each study was determined following a strict combinatorial logic: a study was classified as overall low risk only when all five domains were rated as low risk; if any domain raised some concerns with no domain rated as high risk, the overall judgment was “some concerns”; if any domain was rated as high risk, the study was directly classified as overall high risk. Discrepancies in assessment were resolved through collective discussion or adjudication by a third senior researcher.

### 2.5 Certainty of evidence

The certainty of evidence for core outcome indicators was rated using the GRADE system (18), taking into account five dimensions: risk of bias, inconsistency, indirectness, imprecision, and publication bias. Evidence quality was ultimately classified into four levels: high, moderate, low, and very low. Downgrading decisions were strictly based on the following quantitative and qualitative criteria: downgrading for risk of bias when the overall risk of bias of included studies was high; downgrading for inconsistency when *I*^2^ was substantially elevated and could not be explained through subgroup analysis; and downgrading for imprecision when the 95% CI of the effect size was excessively wide or the total sample size did not meet the optimal information size (OIS).

### 2.6 Data analysis

Quantitative meta-analysis was performed using R software (based on the meta and metafor packages). Given that the measurement units of all outcome indicators were identical, MD and 95% CI were uniformly adopted as effect measures. Data extraction strictly followed the pre-specified protocol, with priority given to change-from-baseline values; where change values were unavailable in the original literature, endpoint values were converted and aligned using standard formulae. Between-study statistical heterogeneity was assessed using the *I*^2^ statistic and τ^2^ value. Given the potential heterogeneity across studies in participant baseline characteristics and exercise protocols, all analyses mandatorily adopted a random-effects model for pooling, with the HKSJ method activated to adjust the pooled variance and control the false-positive rate (19). To explore sources of heterogeneity, hierarchical subgroup analyses were conducted strictly according to control group type (CON vs. AE), with between-subgroup differences evaluated using a χ^2^ test based on the random-effects model. As the number of studies included for each outcome indicator was fewer than 10, sufficient statistical power was not available; therefore, funnel plot and Egger's test for publication bias were not performed.

## 3 Results

### 3.1 Literature screening process

**Figure 1:**
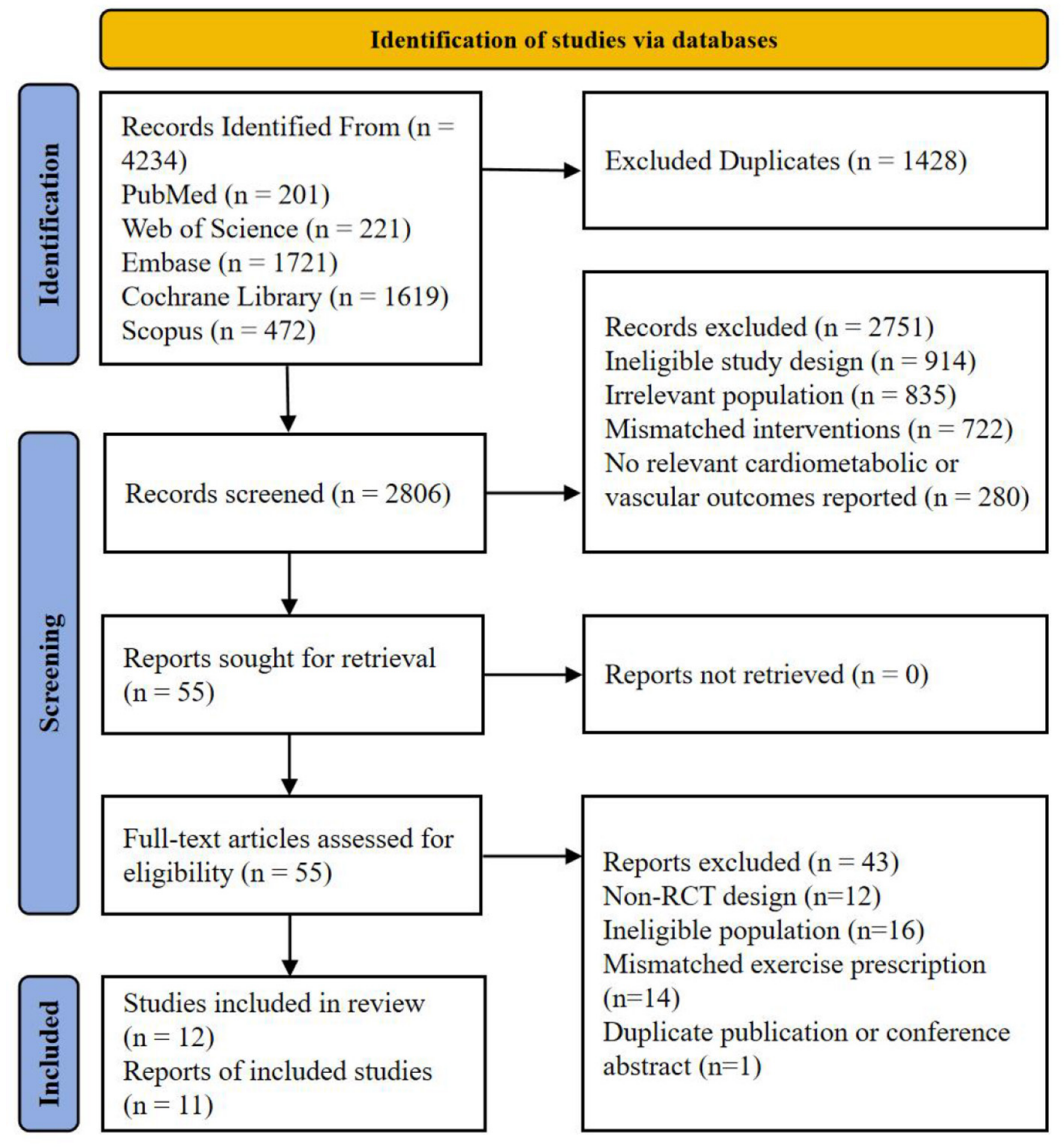
Preferred reporting items for systematic reviews and meta-analysis (PRISMA) study flow diagram.

A total of 4,234 records were retrieved from electronic database searches (PubMed: 201; Web of Science: 221; Embase: 1,721; Cochrane Library: 1,619; Scopus: 472). After removing 1,428 duplicate records using software and manual screening, 2,806 records proceeded to initial screening. Following title and abstract review, 2,751 records were excluded for the following reasons: ineligible study design (*n* = 914), non-target population (*n* = 835), mismatched intervention (*n* = 722), and no relevant cardiometabolic or vascular outcome data reported (*n* = 280). Following initial screening, 55 records with potential eligibility were retrieved for full-text review. After full-text assessment, 43 records were excluded according to the inclusion and exclusion criteria for the following reasons: ineligible study population (*n* = 16), mismatched exercise prescription intervention (*n* = 14), non-randomized controlled design (*n* = 12), and duplicate publication or conference abstract (*n* = 1). Ultimately, 12 RCTs were systematically included in the systematic review. Building on the qualitative synthesis, 1 study that reported only a single set of quantitative data (carotid-femoral pulse wave velocity [cfPWV]) that could not be pooled was excluded from quantitative pooling, leaving 11 RCTs formally entering the quantitative meta-analysis matrix. The detailed screening process and data at each stage are presented in Figure 1.

### 3.2 Basic characteristics of included studies

A total of 12 RCTs were included in this study (20–31), with publication dates spanning from 2011 to 2022 (21, 29). In terms of geographical distribution, more than half of the included trials were conducted in Brazil (*n* = 7) (22–26, 29, 30), followed by the United States (*n* = 3) (20, 21, 31), Canada (*n* = 1) (28), and Iran (*n* = 1) (27). Regarding sample size, the post-randomization sample sizes of the included studies ranged from 30 to 256 participants (20, 28), with the number of participants who completed the study distributed evenly across groups. The age range of participants was 12–19 years (20–31), all of whom were adolescents affected by overweight or obesity [body mass index (BMI) ≥ 95th percentile]; some studies additionally included adolescents with overweight or obesity combined with hyperinsulinemia. In terms of sex composition, the majority of studies enrolled predominantly female participants (accounting for approximately 60%−100%) (20, 21), while some studies included mixed-sex samples. Regarding intervention duration, 5 studies had durations of 8–12 weeks (20–22, 27, 29), 3 studies had durations of 20–24 weeks (23, 28, 31), and 4 studies had durations of 52 weeks (24–26, 30). Training frequency ranged from 2 to 5 sessions per week (20–31), with each session lasting approximately 50–60 mins in total. With respect to the experimental group intervention protocols, aerobic training primarily consisted of moderate-intensity continuous training (MICT) or high-intensity interval training (HIIT), with intensity predominantly set between 40 and 95% of heart rate reserve (HRR), maximum heart rate (HRmax), or maximum velocity (vmax); resistance training consisted primarily of multi-set exercises targeting major muscle groups throughout the body, with intensity predominantly set at 50%−85% of one-repetition maximum (1 RM) or 6–20 RM.

Regarding training sequence in the combined aerobic and resistance training (CART) group, most studies adopted a resistance-before-aerobic sequence, while a small number of studies adopted an aerobic-before-resistance sequence [e.g., Mendonça et al., (29)] or an alternating sequence [e.g., Ahmadi et al., (27); Dâmaso et al., (30)]. With regard to control group design and the comparative framework, to align with the subsequent quantitative hierarchical analysis, the comparator conditions of each study were precisely categorized into two frameworks: 7 studies incorporated a non-exercise control (CON) group [comprising 5 studies with a no-exercise or routine lifestyle control (20–23, 29) and 2 studies with a healthy dietary advice-only group (27, 28)], in which participants did not engage in systematic exercise during the intervention period; 7 studies incorporated an active single-modality aerobic exercise (AE) control group, of which 5 studies with a multi-arm trial design did not include a CON group (24–26, 30, 31) and compared only between different exercise modalities. Regarding outcome indicator reporting, the included studies broadly covered core markers of cardiometabolic and vascular function. In the dimension of glucose metabolism and adipokines, 6 studies reported homeostatic model assessment of insulin resistance (HOMA-IR), and 5 studies reported fasting glucose, fasting insulin, or adiponectin levels, respectively. In the dimension of lipid metabolism, 6 and 5 studies reported total cholesterol (TC) and triglycerides (TG) levels, respectively. In the dimension of vascular function, 5 studies reported trends in systolic blood pressure (SBP) and diastolic blood pressure (DBP), respectively, and only 1 study provided data on subclinical arterial stiffness indicators. Detailed characteristics of each included study and the distribution of outcome indicators are presented in Table 1.

**Table 1:** Basic characteristics of included studies.

References	Sample Size	Age/sex	Health status	Intervention duration	Training frequency	Aerobic/resistance duration (mins)	Intensity	Control group setting	Outcome indicators
Wong et al. (20)	Interventon: 15/Control: 15	15.2 ± 1.2/100% Female	Obese adolescent girls	12 weeks	3 times/week	Resistance before aerobic: 20–30 mins resistance + 30 mins aerobic	Aerobic: 60%−70% HRR; Resistance: 60%−70% 1RM	Non-exercise control	HOMA-IR, TC, TG
Bharath et al. (21)	Interventon: 20/Control: 20	14.6 ± 1/100% Female	Obese with hyperinsulinemia	12 weeks	5 times/week	Resistance before aerobic: 20 mins resistance + 30 mins treadmill aerobic	Aerobic: 40%−70% HRR; Resistance: Moderate intensity	Non-exercise control	Fasting glucose, Adiponectin, TC, SBP
Faria et al. (22)	(1) MICT+RT: 25; (2) HIIT+RT: 26; Control: 25	Approx 16.1/Approx 60% Female	Overweight or obese adolescents	12 weeks	2 times/week	Resistance before aerobic: Aerobic (1) 30; (2) 20; Resistance: Not specified	Aerobic: (1) 50%−60%; (2) 85%−95% HRR; Resistance: 70–85% 1RM	Non-exercise control	Fasting glucose, HOMA-IR, Adiponectin, TC, DBP
Monteiro et al. (23)	(1) Aerobic: 11; (2) Combined: 10; Control: 11	14.3 ± 1.2/Mixed sex	Obese adolescents	20 weeks	3 times/week	(1) 50 mins aerobic; (2) Resistance before aerobic: 30 mins resistance + 20 mins aerobic	Aerobic: 70% VO2peak; Resistance: 10 RM	Non-exercise control	Fasting insulin, DBP
da Silveira Campos et al. (24)	(1) Aerobic: 14; (2) Combined: 16	Approx 16.6/Approx 74% Female	Obese adolescents	52 weeks	3 times/week	(1) 60 mins aerobic; (2) Resistance before aerobic: 30 mins resistance + 30 mins aerobic	Aerobic: Ventilatory threshold 1 (LV1) intensity; Resistance: 50%−70% 1RM	No non-exercise control	Fasting glucose, HOMA-IR, TG, SBP
de Mello et al. (25)	(1) Aerobic: 14; (2) Combined: 16	Approx 16.6/Approx 74% Female	Obese adolescents	52 weeks	3 times/week	(1) 60 mins aerobic; (2) Resistance before aerobic: 30 mins resistance + 30 mins aerobic	Aerobic: Ventilatory threshold 1 (LV1) intensity; Resistance: 50%−70% 1RM	No non-exercise control	Fasting glucose, Fasting insulin, HOMA-IR, TC, TG, SBP, DBP
Campos et al. (26)	(1) Aerobic: 14; (2) Combined: 16	Approx 16.6/Approx 74% Female	Obese adolescents	52 weeks	3 times/week	(1) 60 mins aerobic; (2) Resistance before aerobic: 30 mins resistance + 30 mins aerobic	Aerobic: Ventilatory threshold 1 (LV1) intensity; Resistance: 50%−70% 1RM	No non-exercise control	Fasting glucose, Fasting insulin, Adiponectin, HOMA-IR
Ahmadi et al. (27)	HIIT: 30; RT: 30; Combined: 30; Control: 30	14.41 ± 2.64/53.6% Female	Overweight or obese adolescents	8 weeks	3 times/week	Total 30 mins (including warm-up): Combined group performed alternately	Aerobic: HIIT mode; Resistance: 3 sets, 12 reps/set	Healthy dietary advice alone	Fasting insulin, DBP
Sigal et al. (28)	(1) AE: 62; (2) RE: 66; (3) Combined: 66; Control: 62	15.6 ± 1.4/70.4% Female	Obese adolescents	22 weeks	4 times/week	(1) 45 mins AE; (2) 45 mins RE; (3) 45 mins AE + 45 mins RE	Aerobic: 65%−80% HRmax; Resistance: 2 sets, 8–15 reps	Healthy dietary advice alone	Adiponectin, TC
Mendonça et al. (29)	(1) MICT+RT: 19; (2) HIIT+RT: 20; Control: 24	Approx 15.7/57.9% Female	Brazilian public school adolescents	12 weeks	2 times/week	Aerobic before resistance: Aerobic (1) 15–20; (2) 12–24; Resistance: 8 exercises	Aerobic: 55%−90% vmax; Resistance: 8–20 RM	Routine lifestyle	DBP, SBP
Dâmaso et al. (30)	Combined (AT+RT): 61; Aerobic (AT): 55	16.89 ± 1.81/59% Female	Obese adolescents	52 weeks	3 times/week	(1) 60 mins aerobic; (2) Sequential alternation: 30 mins resistance + 30 mins aerobic	Aerobic: Ventilatory threshold 1 (LV1) intensity; Resistance: 6–20RM	No non-exercise control	Fasting insulin, HOMA-IR, Adiponectin, TC, TG
Lee et al. (31)	(1) AE: 30; (2) RE: 28; (3) Combined: 27	14.4 ± 1.6/64% Female	Overweight or obese adolescents	24 weeks	3 times/week	(1) 60 mins AE; (2) 60 mins RE; (3) 30 mins AE + 30 mins RE	Aerobic: 50%−65% VO2peak; Resistance: 12–15 reps/set	No non-exercise control	TC, TG, DBP, SBP

### 3.3 Risk of bias assessment of included studies

**Figure 2:**
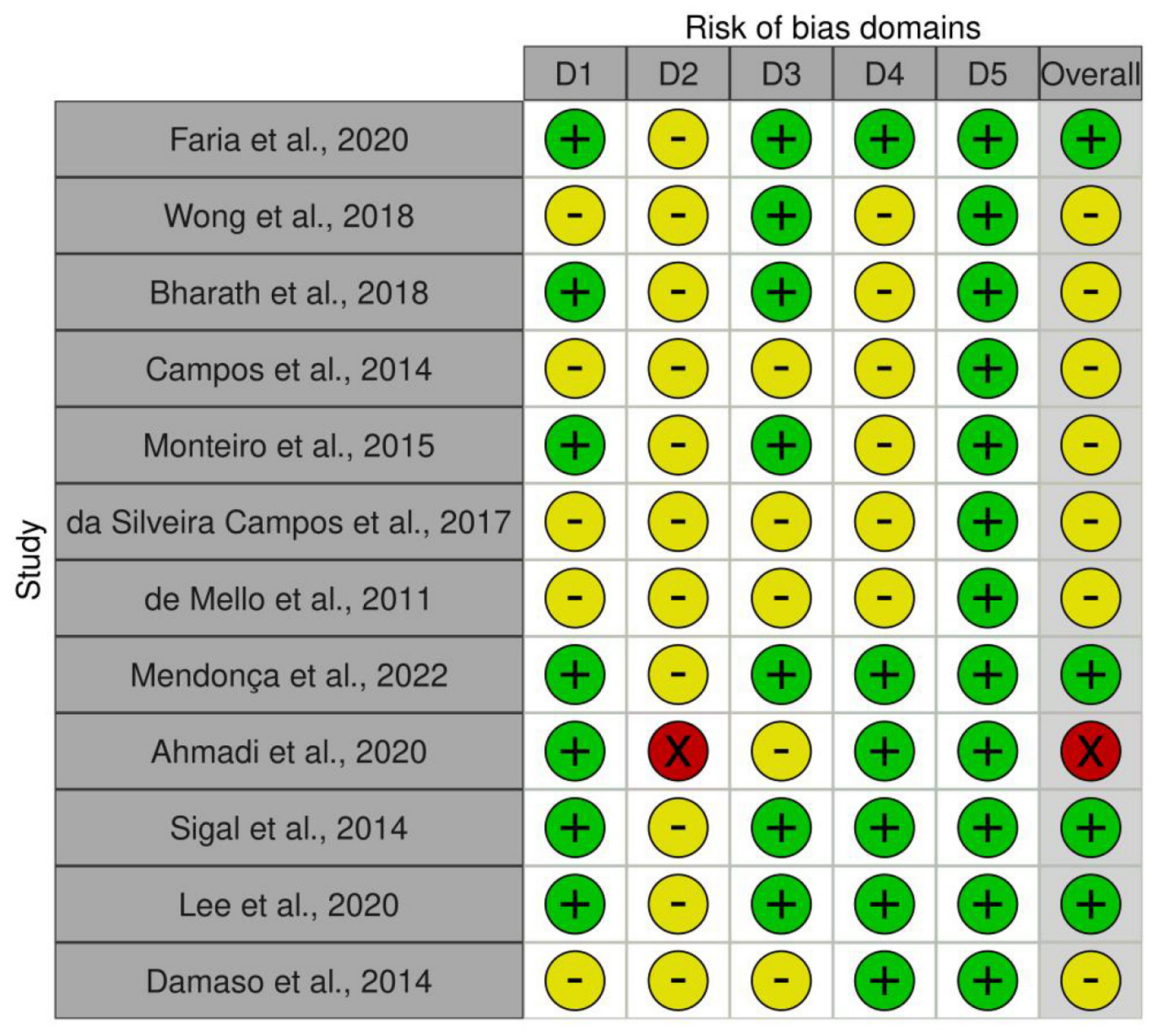
Risk of bias summary: review of the authors judgments about each risk of bias item for each included study.

**Figure 3:**
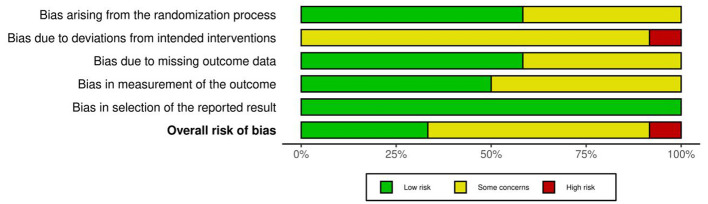
Risk of bias graph: review authors' judgments about each risk of bias item, presented as percentage of included studies.

A total of 12 RCTs were assessed for methodological quality using RoB 2.0 (see Figures 2, 3). Overall results showed that 4 studies were rated as low risk of bias; 7 studies were rated as “some concerns” due to the inability to blind participants to the exercise intervention (Domain 2) (32) or insufficient reporting of allocation concealment and outcome assessment blinding (Domains 1 and 4); and 1 study Ahmadi et al. (27) was rated as high risk of bias due to insufficient supervision of home-based training leading to low intervention fidelity. Through cross-domain identification, “Domain 2: bias due to deviations from the intended intervention” and “Domain 1: inadequate disclosure of allocation concealment in the randomization process” were identified as the primary sources of concern for risk of bias in the literature of this field.

From a domain-specific perspective: Domain 2 (deviations from intended interventions) was the domain with the most concentrated risk (32); with the exception of the majority of studies that maintained high fidelity through on-site supervision, only Ahmadi et al. (27) was rated as high risk due to home-based telephone follow-up that could not verify intervention fidelity. Domain 1 (randomization process) was another core source of “some concerns” ratings, with 5 studies Wong et al. (20), Campos et al. (26), da Silveira Campos et al. (24), de Mello et al. (25), and Dâmaso et al. (30) all downgraded due to insufficient description of allocation concealment. In contrast, the included studies performed well overall across Domain 3 (missing outcome data), Domain 4 (measurement of outcomes), and Domain 5 (selection of reported results), with most studies demonstrating low dropout rates [or employing intention-to-treat (ITT) analysis], explicitly reporting assessor blinding, and providing clinical trial registration information.

### 3.4 Meta-analysis results

#### 3.4.1 Lipid indicators

Regarding total cholesterol (TC), a total of 6 studies were included (experimental group: 207 participants; control group: 175 participants). The overall random-effects model analysis showed that the effect of combined exercise intervention on TC did not reach statistical significance (MD = −3.48 mg/dL, 95% CI: −10.12 to 3.16, *P* = 0.30, *I*^2^ = 41.00%, P_Q = 0.13). Subgroup analysis by control group type showed that neither subgroup reached statistical significance: in the CART vs. non-exercise control subgroup (*k* = 3, 93/69 participants), MD = −1.79 mg/dL (95% CI: −12.39 to 8.80, *P* = 0.74, *I*^2^ = 5.70%, P_Q = 0.35); in the CART vs. aerobic exercise subgroup (*k* = 3, 114/106 participants), MD = −5.51 mg/dL (95% CI: −17.77 to 6.74, *P* = 0.38, *I*^2^ = 68.20%, P_Q = 0.04). The difference between the two subgroups was not statistically significant (P_between = 0.65) (see Supplementary Figure 1).

Regarding triglycerides (TG), a total of 5 studies were included (experimental group: 157 participants; control group: 149 participants). The overall random-effects model analysis showed that the pooled result did not reach statistical significance (MD = 3.90 mg/dL, 95% CI: −4.92 to 12.72, *P* = 0.39, *I*^2^ = 26.50%, P_Q = 0.24). Subgroup analysis showed that the effect sizes in both subgroups were in opposite directions and neither reached statistical significance: in the CART vs. non-exercise control subgroup (*k* = 2, 43/43 participants), MD = −8.08 mg/dL (95% CI: −28.87 to 12.72, *P* = 0.45, *I*^2^ = 0.00%, P_Q = 0.93); in the CART vs. aerobic exercise subgroup (*k* = 3, 114/106 participants), MD = 8.17 mg/dL (95% CI: −5.03 to 21.37, *P* = 0.22, I^2^ = 48.40%, P_Q = 0.14). The difference between the two subgroups was not statistically significant (P_between = 0.20) (see Supplementary Figure 2). The pooled results are presented in Table 2.

**Table 2:** Meta-analysis and subgroup analysis results of the effects of combined exercise intervention on lipid indicators in adolescents with overweight or obesity.

Outcome indicator	Subgroup/full model	Number of studies (*k*)	Sample size (E/C)	Mean difference (MD)	95% Confidence interval (95% CI)	Significance of effect size (*P*)	Heterogeneity *I^2^* (%)	Heterogeneity test (P_Q_)	Test for subgroup differences (P_between_)
TC (mg/dL)	Full random-effects model	6	207/175	−3.48	(−10.12, 3.16)	0.3	41.00%	0.13	—
	CART vs. CON	3	93/69	−1.79	(−12.39, 8.80)	0.74	5.70%	0.35	0.65
	CART vs. AE	3	114/106	−5.51	(−17.77, 6.74)	0.38	68.20%	0.04	
TG (mg/dL)	Full random-effects model	5	157/149	3.9	(−4.92, 12.72)	0.39	26.50%	0.24	—
	CART vs. CON	2	43/43	−8.08	(−28.87, 12.72)	0.45	0.00%	0.93	0.2
	CART vs. AE	3	114/106	8.17	(−5.03, 21.37)	0.22	48.40%	0.14	

TC, total cholesterol; TG, triglycerides; E, experimental group (CART).

#### 3.4.2 Blood pressure indicators

Regarding systolic blood pressure (SBP), a total of 5 studies were included (experimental group: 198 participants; control group: 173 participants). The overall random-effects model analysis showed that the overall effect size did not reach statistical significance (MD = −2.50 mmHg, 95% CI: −5.22 to 0.22, *P* = 0.07, *I*^2^ = 60.30%, P_Q = 0.04). Subgroup analysis showed that in the CART vs. non-exercise control subgroup (*k* = 2, 70/46 participants), the intervention effect reached statistical significance (MD = −3.51 mmHg, 95% CI: −6.23 to −0.80, *P* = 0.01, *I*^2^ = 0.00%, P_Q = 0.67); whereas in the CART vs. aerobic exercise subgroup (*k* = 3, 128/127 participants), the effect did not reach statistical significance (MD = −3.23 mmHg, 95% CI: −10.42 to 3.96, *P* = 0.38, *I*^2^ = 73.30%, P_Q < 0.01). The difference between the two subgroups was not statistically significant (P_between = 0.94) (see Supplementary Figure 3).

Regarding diastolic blood pressure (DBP), a total of 5 studies were included (experimental group: 198 participants; control group: 173 participants). The overall random-effects model analysis showed that the overall effect size did not reach statistical significance (MD = −1.27 mmHg, 95% CI: −3.53 to 0.99, *P* = 0.27, *I*^2^ = 47.00%, P_Q = 0.11). Subgroup analysis showed that in the CART vs. non-exercise control subgroup (*k* = 2, 70/46 participants), the intervention effect reached statistical significance (MD = −3.72 mmHg, 95% CI: −6.69 to −0.75, *P* = 0.01, *I*^2^ = 0.00%, P_Q = 0.47); whereas in the CART vs. aerobic exercise subgroup (*k* = 3, 128/127 participants), the intervention effect did not reach statistical significance (MD = 0.15 mmHg, 95% CI: −1.59 to 1.88, *P* = 0.87, *I*^2^ = 8.20%, P_Q = 0.34). The difference between the two subgroups was statistically significant (P_between = 0.03), indicating that control group type has a substantive moderating effect on the estimation of the effect size for DBP. Additionally, in an individual study, Mendonça et al. (29) reported that following 12 weeks of intervention, both moderate-intensity continuous training combined with resistance training and HIIT combined with resistance training showed a positive downward trend in both SBP and DBP among participants. The pooled blood pressure results are presented in Table 3.

**Table 3:** Meta-analysis and subgroup analysis results of the effects of combined exercise intervention on vascular function (blood pressure) in adolescents with overweight or obesity.

Outcome indicator	Subgroup/full model	Number of studies (k)	Sample size (E/C)	Mean difference (MD)	95% Confidence interval (95% CI)	Significance of effect size (*P*)	Heterogeneity *I^2^* (%)	Heterogeneity test (P_Q_)	Test for subgroup differences (P_between_)
SBP (mmHg)	Full random-effects model	5	198/173	−2.5	(−5.22, 0.22)	0.07	60.30%	0.04	—
	CART vs. CON	2	70/46	−3.51	(−6.23, −0.80)	0.01	0.00%	0.67	0.94
	CART vs. AE	3	128/127	−3.23	(−10.42, 3.96)	0.38	73.30%	<0.01	
DBP (mmHg)	Full random-effects model	5	198/173	−1.27	(−3.53, 0.99]	0.27	47.00%	0.11	—
	CART vs. CON	2	70/46	−3.72	(−6.69, −0.75)	0.01	0.00%	0.47	0.03
	CART vs. AE	3	128/127	0.15	(−1.59, 1.88)	0.87	8.20%	0.34	

SBP, systolic blood pressure; DBP, diastolic blood pressure.

#### 3.4.3 Glucose metabolism and adipokine indicators

Regarding adiponectin, a total of 5 studies were included (experimental group: 180 participants; control group: 150 participants). The overall random-effects model analysis showed that the overall effect size reached statistical significance (MD = 2.22 μg/mL, 95% CI: 1.47 to 2.97, *P* < 0.0001, *I*^2^ = 43.50%, P_Q = 0.13). Subgroup analysis showed that in the CART vs. non-exercise control subgroup (*k* = 1, 20/20 participants), the result did not reach statistical significance (MD = 1.30 μg/mL, 95% CI: −0.45 to 3.05, *P* = 0.15); whereas in the CART vs. aerobic exercise subgroup (*k* = 4, 160/130 participants), the intervention effect reached statistical significance (MD = 2.37 μg/mL, 95% CI: 1.53 to 3.21, *P* < 0.0001, *I*^2^ = 49.80%, P_Q = 0.11). The difference between the two subgroups was not statistically significant (P_between = 0.28) (see Supplementary Figure 5).

Regarding homeostatic model assessment of insulin resistance (HOMA-IR), a total of 6 studies were included (experimental group: 230 participants; control group: 176 participants). The overall random-effects model analysis showed that the overall effect size did not reach statistical significance (MD = −1.10, 95% CI: −2.47 to 0.28, *P* = 0.12, *I*^2^ = 86.70%, P_Q < 0.0001). Subgroup analysis showed that in the CART vs. non-exercise control subgroup (*k* = 2, 70/46 participants), the result did not reach statistical significance (MD = −2.24, 95% CI: −7.34 to 2.85, *P* = 0.39, *I*^2^ = 97.20%, P_Q < 0.0001), with extremely high heterogeneity; whereas in the CART vs. aerobic exercise subgroup (*k* = 4, 160/130 participants), the effect reached statistical significance (MD = −0.57, 95% CI: −1.01 to −0.12, *P* = 0.01, *I*^2^ = 0.00%, P_Q = 0.98). The difference between the two subgroups was not statistically significant (P_between = 0.52) (see Supplementary Figure 6).

Regarding fasting insulin, a total of 5 studies were included (experimental group: 175 participants; control group: 145 participants). The overall random-effects model analysis showed that the overall effect size reached statistical significance (MD = −5.43 μU/mL, 95% CI: −10.32 to −0.55, *P* = 0.03, *I*^2^ = 72.20%, P_Q = 0.01). Subgroup analysis showed that both subgroups reached statistical significance: in the CART vs. non-exercise control subgroup (*k* = 1, 15/15 participants), MD = −17.90 μU/mL (95% CI: −26.04 to −9.76, *P* < 0.0001); in the CART vs. aerobic exercise subgroup (*k* = 4, 160/130 participants), MD = −2.70 μU/mL (95% CI: −4.61 to −0.79, *P* = 0.01, *I*^2^ = 0.00%, P_Q = 0.79). The difference between the two subgroups was statistically significant (P_between = 0.0004), indicating that control group type has a substantive moderating effect on the estimation of the insulin effect size (see Supplementary Figure 7).

Regarding fasting glucose, a total of 5 studies were included (experimental group: 164 participants; control group: 116 participants). The overall random-effects model analysis showed that the overall effect size did not reach statistical significance (MD = −5.48 mg/dL, 95% CI: −12.66 to 1.69, *P* = 0.13, *I*^2^ = 98.40%, P_Q < 0.0001). Subgroup analysis showed that neither subgroup reached statistical significance and both exhibited extremely high heterogeneity: in the CART vs. non-exercise control subgroup (*k* = 2, 65/41 participants), MD = −9.60 mg/dL (95% CI: −26.23 to 7.03, *P* = 0.26, *I*^2^ = 98.30%, P_Q < 0.0001); in the CART vs. aerobic exercise subgroup (*k* = 3, 99/75 participants), MD = −2.57 mg/dL (95% CI: −8.70 to 3.56, *P* = 0.41, *I*^2^ = 89.20%, P_Q < 0.0001). The difference between the two subgroups was not statistically significant (P_between = 0.44) (see Supplementary Figure 8). The pooled results are presented in Table 4. Additionally, Lee et al. (31) is the only study in this review to have reported data on the subclinical atherosclerosis marker carotid-femoral pulse wave velocity (cfPWV). Following 24 weeks of intervention, the change in cfPWV in the combined training group was +1.5 cm/s (SE = 14.9, *P* = 0.92), while in the aerobic exercise group it was −18.5 cm/s (SE = 13.9, *P* = 0.18), with the between-group difference in change also not reaching statistical significance (31).

**Table 4:** Meta-analysis and subgroup analysis results of the effects of combined exercise intervention on glucose metabolism and chronic inflammation indicators in adolescents with overweight or obesity.

Outcome indicator	Subgroup/full model	Number of studies (k)	Sample size (E/C)	Mean difference (MD)	95% Confidence interval (95% CI)	Significance of effect size (*P*)	Heterogeneity *I^2^* (%)	Heterogeneity test (P_Q_)	Test for subgroup differences (P_between_)
Adiponectin (μg/mL)	Full random-effects model	5	180/150	2.22	(1.47, 2.97)	<0.0001	43.50%	0.13	—
	CART vs. CON	1	20/20	1.3	(−0.45, 3.05)	0.15	—	—	0.28
	CART vs. AE	4	160/130	2.37	(1.53, 3.21)	<0.0001	49.80%	0.11	
HOMA-IR	Full random-effects model	6	230/176	−1.1	(−2.47, 0.28)	0.12	86.70%	<0.0001	—
	CART vs. CON	2	70/46	−2.24	(−7.34, 2.85]	0.39	97.20%	<0.0001	0.52
	CART vs. AE	4	160/130	−0.57	(−1.01, −0.12)	0.01	0.00%	0.98	
Insulin (μU/mL)	Full random-effects model	5	175/145	−5.43	(−10.32, −0.55)	0.03	72.20%	0.01	—
	CART vs. CON	1	15/15	−17.9	(−26.04, −9.76)	<0.0001	—	—	0.0004
	CART vs. AE	4	160/130	−2.7	(−4.61, −0.79)	0.01	0.00%	0.79	
Fasting Glucose (mg/dL)	Full random-effects model	5	164/116	−5.48	(−12.66, 1.69)	0.13	98.40%	<0.0001	—
	CART vs. CON	2	65/41	−9.6	(−26.23, 7.03)	0.26	98.30%	<0.0001	0.44
	CART vs. AE	3	99/75	−2.57	(−8.70, 3.56)	0.41	89.20%	<0.0001	

HOMA-IR = homeostatic model assessment of insulin resistance.

### 3.5 Evidence quality assessment

The GRADE system was used to rate the certainty of evidence for core outcome indicators. As exercise interventions cannot be blinded to participants and implementers, and as allocation concealment was insufficiently described in some studies, all indicators were uniformly downgraded by one level on the risk of bias dimension; as the number of studies included for each indicator was fewer than 10 and funnel plots were not constructed, publication bias was rated as “not assessed” for all indicators; no downgrading was applied for indirectness for any indicator. In the CART vs. non-exercise control subgroup (see Table 5), fasting insulin, systolic blood pressure (SBP), and diastolic blood pressure (DBP) were ultimately rated as “moderate” certainty of evidence, as there was no heterogeneity within the subgroup (*I*^2^ = 0.0%) and the 95% CI was relatively narrow and did not cross the null axis. Adiponectin, total cholesterol (TC), and triglycerides (TG) were ultimately rated as “low,” as although heterogeneity was acceptable (*I*^2^ ≤ 5.7%), imprecision was downgraded by one level due to the 95% CI crossing the null axis. Fasting glucose and homeostatic model assessment of insulin resistance (HOMA-IR) were ultimately rated as “very low,” as extremely severe clinical and statistical heterogeneity within the subgroup (*I*^2^ = 98.3% and *I*^2^ = 97.2%, respectively) warranted a two-level downgrade for inconsistency, and the extremely wide CI crossing the null axis warranted a further one-level downgrade for imprecision.

**Table 5:** GRADE evidence rating for the CART vs. non-exercise control subgroup.

Outcome indicator	Risk of bias	Inconsistency	Indirectness	Imprecision	Publication bias	Quality of evidence	Grade
Fasting Glucose	Downgraded by 1 level	Downgraded by 2 levels (*I^2^*= 98.3%)	None	Downgraded by 1 level (extremely wide CI crossing zero)	Not evaluated	Very low	⊕◯◯◯
Fasting Insulin	Downgraded by 1 level	Not downgraded (*k* = 1)	None	Not downgraded (CI did not cross zero)	Not evaluated	Moderate	⊕⊕⊕◯
HOMA-IR	Downgraded by 1 level	Downgraded by 2 levels (*I^2^* = 97.2%)	None	Downgraded by 1 level (extremely wide CI crossing zero)	Not evaluated	Very low	⊕◯◯◯
Adiponectin	Downgraded by 1 level	Not downgraded (*k* = 1)	None	Downgraded by 1 level (CI crossed zero)	Not evaluated	Low	⊕⊕◯◯
TC	Downgraded by 1 level	Not downgraded (*I^2^* = 5.7%)	None	Downgraded by 1 level (CI crossed zero)	Not evaluated	Low	⊕⊕◯◯
TG	Downgraded by 1 level	Not downgraded (*I^2^* = 0.0%)	None	Downgraded by 1 level (CI crossed zero)	Not evaluated	Low	⊕⊕◯◯
SBP	Downgraded by 1 level	Not downgraded (*I^2^* = 0.0%)	None	Not downgraded (CI did not cross zero)	Not evaluated	Moderate	⊕⊕⊕◯
DBP	Downgraded by 1 level	Not downgraded (*I^2^* = 0.0%)	None	Not downgraded (CI did not cross zero)	Not evaluated	Moderate	⊕⊕⊕◯

Grade symbols indicate the quality of evidence: ⊕⊕⊕⊕ High quality; ⊕⊕⊕ Moderate quality; ⊕⊕ Low quality; ⊕ Very low quality.

In the CART vs. aerobic exercise subgroup (see Table 6), fasting insulin and HOMA-IR were ultimately rated as “moderate” certainty of evidence, as both demonstrated complete consistency within the subgroup (*I*^2^ = 0.0%) and the CI did not cross the null axis. Adiponectin was downgraded by one level for inconsistency due to moderate heterogeneity (*I*^2^ = 49.8%); TC (*I*^2^ = 68.2%) and TG (*I*^2^ = 48.4%) were similarly downgraded by one level for inconsistency due to relatively high heterogeneity, and further downgraded by one level for imprecision due to the CI crossing the null axis; DBP was downgraded by one level for imprecision due to the CI crossing the null axis (*I*^2^ = 8.2%). The certainty of evidence for these four indicators was ultimately rated as “low.” SBP was downgraded by two levels for inconsistency due to substantial heterogeneity (*I*^2^ = 73.3%) and by one further level for imprecision due to the CI crossing the null axis; fasting glucose was similarly downgraded by two levels for inconsistency due to extremely high heterogeneity (*I*^2^ = 89.2%) and by one further level for imprecision due to the extremely wide CI crossing the null axis. The certainty of evidence for both indicators was ultimately rated as “very low.” The specific rating scores and judgment details for each included study are presented in Table 6.

**Table 6:** GRADE evidence rating for the CART vs. aerobic exercise subgroup.

Outcome indicator	Risk of bias	Inconsistency	Indirectness	Imprecision	Publication bias	Quality of evidence	Grade
Fasting Glucose	Downgraded by 1 level	Downgraded by 2 levels (*I^2^* = 89.2%)	None	Downgraded by 1 level (extremely wide CI crossing zero)	Not evaluated	Very low	⊕◯◯◯
Fasting Insulin	Downgraded by 1 level	Not downgraded (*I^2^* = 0.0%)	None	Not downgraded (CI did not cross zero)	Not evaluated	Moderate	⊕⊕⊕◯
HOMA-IR	Downgraded by 1 level	Not downgraded (*I^2^* = 0.0%)	None	Not downgraded (CI did not cross zero)	Not evaluated	Moderate	⊕⊕⊕◯
Adiponectin	Downgraded by 1 level	Downgraded by 1 level (*I^2^* = 49.8%)	None	Not downgraded (CI did not cross zero)	Not evaluated	Low	⊕⊕◯◯
TC	Downgraded by 1 level	Downgraded by 1 level (*I^2^* = 68.2%)	None	Downgraded by 1 level (CI crossed zero)	Not evaluated	Low	⊕⊕◯◯
TG	Downgraded by 1 level	Downgraded by 1 level (*I^2^* = 48.4%)	None	Downgraded by 1 level (CI crossed zero)	Not evaluated	Low	⊕⊕◯◯
SBP	Downgraded by 1 level	Downgraded by 2 levels (*I^2^* = 73.3%)	None	Downgraded by 1 level (CI crossed zero)	Not evaluated	Very low	⊕◯◯◯
DBP	Downgraded by 1 level	Not downgraded (*I^2^* = 8.2%)	None	Downgraded by 1 level (CI crossed zero)	Not evaluated	Low	⊕⊕◯◯

Grade symbols indicate the quality of evidence: ⊕⊕⊕⊕ High quality; ⊕⊕⊕ Moderate quality; ⊕⊕ Low quality; ⊕ Very low quality.

## 4 Discussion

### 4.1 Summary of findings

In the comparison of CART vs. non-exercise control, fasting insulin, systolic blood pressure (SBP), and diastolic blood pressure (DBP) all reached statistical significance, and as the within-subgroup statistical heterogeneity for all three indicators was 0%, the results demonstrated good internal consistency; total cholesterol (TC), triglycerides (TG), and adiponectin did not reach statistical significance; fasting glucose and homeostatic model assessment of insulin resistance (HOMA-IR) exhibited extremely wide confidence intervals due to extremely high within-subgroup heterogeneity (*I*^2^ > 97%), and the available limited data carry considerable uncertainty. In the comparison of CART vs. aerobic exercise, fasting insulin and HOMA-IR reached statistical significance, and the within-subgroup heterogeneity for both indicators was 0%, displaying extremely low statistical heterogeneity; adiponectin reached statistical significance (*I*^2^ = 49.8%); SBP, TC, TG, DBP, and fasting glucose did not reach statistical significance, with SBP showing relatively high heterogeneity (*I*^2^ = 73.3%) and fasting glucose showing extremely high heterogeneity (*I*^2^ = 89.2%), suggesting that the relevant outcomes require cautious interpretation in light of clinical realities. Furthermore, statistically significant between-subgroup differences in effect sizes were observed for fasting insulin and DBP across the two subgroups (*P* = 0.0004; *P* = 0.03), indicating that control group type exerts a substantive moderating effect on effect size estimation, further confirming the methodological necessity of the dual-subgroup design. Regarding evidence quality, only specific indicators in certain subgroups such as fasting insulin and blood pressure in the CART vs. non-exercise control subgroup, and fasting insulin and HOMA-IR in the CART vs. aerobic exercise subgroup reached moderate certainty of evidence, while the certainty for most remaining indicators remained low or very low. This is primarily constrained by the inherent inability to blind exercise interventions, relatively high clinical heterogeneity in some subgroups, and limited sample sizes of included studies; therefore, the overall conclusions still require targeted validation in future large-sample, high-quality studies.

### 4.2 Comparison with prior research and causal analysis

The effect directions of each indicator in this study are broadly consistent with the existing literature, although the statistical significance of some indicators has changed to a certain extent due to the addition of new study data. The significant reduction in fasting insulin in the CART vs. non-exercise control comparison is directionally consistent with Marson et al. (13), who demonstrated that exercise training as a whole can significantly reduce fasting insulin; HOMA-IR reached statistical significance in the CART vs. aerobic exercise subgroup with *I*^2^ = 0%, corroborating the conclusion of Marson et al. (13) that exercise training can improve HOMA-IR. This also partially explains why that review noted that the comparative effects between different exercise modalities remain controversial; the present study further suggests that this controversy may largely stem from the conflation of control group types (13). Adiponectin reached statistical significance in the CART vs. aerobic exercise subgroup, directionally consistent with García-Hermoso et al. (11), who noted that the benefits of CART on adiponectin are particularly prominent when intervention duration exceeds 24 weeks; the fact that adiponectin still reached significance despite the relatively short intervention periods of the included studies in this review suggests a certain degree of trend stability for this effect. Regarding lipids, TC in both subgroups was directionally consistent with Chen et al. (12) but neither reached statistical significance; the negative direction of TG in the CART vs. non-exercise control subgroup is consistent with Chen et al. (12), while the positive direction in the CART vs. aerobic exercise subgroup, given the clinical uncertainty of this subgroup's results, is not suitable for direct absolutized comparison with prior research (12). Regarding blood pressure, SBP and DBP both reached statistical significance in the CART vs. non-exercise control subgroup, directionally consistent with existing exercise intervention literature (33); neither reached significant effects in the CART vs. aerobic exercise subgroup, and the high SBP heterogeneity observed (*I*^2^ = 73.3%) as a *post-hoc* methodological interpretation may partially be associated with between-group baseline imbalances in Sigal et al. (28) and de Mello et al. (25), although in the absence of independent sensitivity analysis support, this causal explanation remains speculative (25, 28). Regarding arterial stiffness, all three groups in Lee et al. (31) showed no significant improvement in cfPWV, consistent with the conclusion of Sequi-Dominguez et al. (34) that combined exercise effects did not reach significance in their network meta-analysis.

From the perspective of biological mechanisms and statistical causation, fasting insulin reached statistical significance in both subgroups, but with a substantive difference in effect size. The effect size in the CART vs. non-exercise control subgroup was considerably larger than in the CART vs. aerobic exercise subgroup; the former reflects the absolute benefit of CART compared with no exercise at all, while the latter suggests that CART may produce additional insulin-lowering effects on top of aerobic training. The physiological basis for this lies in aerobic exercise activating the AMPK pathway to mediate GLUT4 translocation and accelerate glucose clearance (35), while resistance training induces skeletal muscle hypertrophy through the mTOR pathway to expand peripheral glucose storage capacity; the synergistic effect of the two may exceed that of a single exercise modality, and the consistent significance across both subgroups provides relatively consistent preliminary corroboration for CART's improvement of insulin metabolism (36). The effective reduction in fasting insulin may help relieve the inhibitory effect of hyperinsulinemia on hormone-sensitive lipase, potentially reverse the vicious cycle of “hyperinsulinemia-fat accumulation,” and reduce the compensatory overload on pancreatic β cells; this clinically suggests the potential long-term implications (potential implications) of CART in preventing or delaying the progression from overweight or obesity to type 2 diabetes in adolescents, although the definitive long-term protective benefits still require tracking and confirmation in future studies with longer follow-up periods (37, 38). HOMA-IR reached statistical significance with *I*^2^ = 0% in the CART vs. aerobic exercise subgroup, while the CART vs. non-exercise control subgroup exhibited extremely high heterogeneity (*I*^2^ = 97.2%) (21); we speculate that this may be partly attributable to clinical heterogeneity between the individualized intensity setting adopted by Bharath et al. (21) and the fixed-intensity settings used in other protocols. The striking contrast between the two subgroups suggests a potential moderating effect of intervention protocol standardization on the stability of results, but in the absence of formal subgroup verification, this view should be explicitly framed as a speculative *post-hoc* interpretation. Because the 4 studies in the CART vs. aerobic exercise subgroup demonstrated relatively good homogeneity due to their similar fixed-intensity settings, with sufficient statistical power, allowing the effect to emerge stably. Both subgroups for fasting glucose failed to reach significance and exhibited extremely high heterogeneity; *post-hoc* clinical analysis suggests that this may primarily tend to reflect the population baseline differences between Wong et al. (20), who enrolled adolescents with type 1 diabetes mellitus (T1DM), and Faria et al. (22), who enrolled generally overweight adolescents with normal baseline glucose levels, with the two populations having different margins for improvement; as no sensitivity analysis targeting population type was conducted, this baseline difference explanation serves only as a *post-hoc* inference, and therefore direct pooled analysis currently carries considerable uncertainty.

Adiponectin in the CART vs. non-exercise control subgroup included only a single study (*k* = 1), with extremely limited statistical power, and did not reach significance; in the CART vs. aerobic exercise subgroup, the accumulation of 4 studies with highly consistent effect directions substantially improved statistical power, ultimately reaching significance. Adiponectin assists in improving insulin resistance through enhancing fatty acid oxidation and reducing hepatic glucose output, and holds important potential value for improving the chronic low-grade inflammatory state in adolescents (39, 40). SBP and DBP both reached statistical significance in the CART vs. non-exercise control subgroup with *I*^2^ = 0%, suggesting that compared with no exercise, CART's improvement of blood pressure demonstrates relatively good internal consistency; neither reached statistical significance in the CART vs. aerobic exercise subgroup, and as a reasonable *post-hoc* speculation, the statistical variability may be partially associated with the notable between-group baseline imbalances in de Mello et al. (25) and Sigal et al. (28), with baseline differences potentially objectively diluting the true intervention effect, suggesting that this may serve as one potential *post-hoc* explanatory source for SBP heterogeneity rising to 73.3%. The statistically significant between-subgroup difference in DBP (*P* = 0.03) further confirms that the activity level of the control group is a key source of influence on blood pressure effect size estimation, suggesting its potential clinical implications in interrupting the chain of progression from elevated blood pressure in adolescence to primary hypertension and early vascular damage in adulthood. It must be noted, however, that this extension to long-term outcomes is currently only a speculative potential implication, as it has not been directly confirmed given the short follow-up periods of the RCT evidence in this batch, and future multicenter prospective cohort studies with longer follow-up periods are still needed for systematic confirmation (41, 42). The two subgroups for TG showed opposite directions; the negative direction in the CART vs. non-exercise control subgroup is consistent with existing literature, while the positive direction in the CART vs. aerobic exercise subgroup is potentially associated from a methodological standpoint with the limited data precision arising from median-derived estimation and extremely small sample sizes in de Mello et al. (25). Given that this causal exploration lacks quantitative support from independent sensitivity or subgroup analyses, it does not necessarily reflect a true physiological effect and constitutes a speculative *post-hoc* interpretation; therefore, absolute substantive conclusions should not be drawn on this basis. Regarding arterial stiffness, Lee et al. (31) as the current sole single-study evidence cannot be subjected to quantitative pooling and represents a critical evidence gap urgently awaiting resolution in this field.

### 4.3 Study limitations

This study has the following limitations. First, heterogeneity among included studies was generally high, with the extremely high heterogeneity of fasting glucose and HOMA-IR (*I*^2^ > 90%) rendering the corresponding subgroup results unreliable, primarily attributable to differences in population baseline metabolic status and inconsistencies in the method of exercise intensity prescription. Second, the nature of exercise interventions precludes blinding of participants, and all included studies carry a risk of systematic bias arising from this constraint, which is an inherent limitation of research design in this field (32). Third, the intervention periods of included studies were generally short, which may have led to an underestimation of indicators such as adiponectin that require longer periods to manifest effects. Fourth, data on arterial stiffness indicators (cfPWV) were provided by only a single study, precluding quantitative pooling, and the available evidence is extremely limited. Fifth, the number of studies included for each indicator was fewer than 10, making it impossible to construct funnel plots for quantitative assessment of publication bias, and the risk of publication bias cannot be excluded (43). Sixth, considerable variability existed across studies in exercise intensity, frequency, duration, and the proportional allocation of aerobic and resistance training, limiting the generalizability of the findings. Seventh, the geographical distribution of included trials was severely imbalanced, with the vast majority highly concentrated in Brazil and North America, which creates a critical research gap when extrapolating the efficacy of CART to broader global populations. Eighth, the search strategy of this study excluded gray literature, dissertations, and preprint sources, which may have resulted in the omission of some unpublished studies with negative results; furthermore, as the number of studies included for each indicator was fewer than 10, quantitative assessment of publication bias through funnel plots was not feasible, and therefore this study carries a potential risk of publication bias.

### 4.4 Practical implications

Given the variability in exercise intensity, frequency, duration, and modality allocation across included studies, the generalizability of the conclusions of this study to clinical and physical education practice is limited (limited generalizability), and these findings cannot serve as standardized exercise prescription guidelines, but only as the following qualified reference points: First, fasting insulin reached statistical significance in both subgroups, suggesting that whether compared with no exercise or aerobic exercise alone, CART can reduce fasting insulin levels in adolescents with overweight or obesity, and may serve as a non-pharmacological exploratory approach for adolescents with hyperinsulinemia. Second, HOMA-IR reached statistical significance in the CART vs. aerobic exercise subgroup with *I*^2^ = 0%, suggesting that combined resistance training may possess a certain degree of incremental benefit over aerobic exercise alone; however, in the context of differing exercise intensity settings across trials, this only suggests that the selective combination of individualized resistance training on top of conventional aerobic exercise may be worth considering (44). Third, adiponectin reached statistical significance in the CART vs. aerobic exercise subgroup, suggesting that CART may be superior to aerobic exercise alone in elevating adiponectin and improving chronic inflammation, although the optimal exercise frequency remains to be standardized. Fourth, SBP and DBP both reached statistical significance in the CART vs. non-exercise control subgroup with heterogeneity of 0%, suggesting that within the constrained exercise dose ranges, CART demonstrates potential application prospects for improving blood pressure. In summary, due to the heterogeneity in exercise dose parameters, the above practice-oriented insights must be accompanied by strict clinical qualifications, and their application is of reference value only within the specific exercise dose ranges comparable to those of the included studies. The available evidence is insufficient to support the formulation of a standardized universal CART exercise prescription, and further randomized controlled trials with standardized dosing and adequate sample sizes are still needed for confirmation.

## 5 Conclusion

This study evaluated the effects of CART on cardiometabolic indicators in adolescents with overweight or obesity through a dual-subgroup design. In the CART vs. non-exercise control subgroup, fasting insulin and blood pressure indicators reached statistical significance, suggesting that compared with no exercise, CART demonstrates potential application prospects for improving insulin metabolism and blood pressure levels, although the actual clinical meaningfulness remains constrained by the low certainty of the available evidence. In the CART vs. aerobic exercise subgroup, fasting insulin, HOMA-IR, and adiponectin reached statistical significance, suggesting that compared with aerobic exercise alone, CART may possess a certain degree of incremental benefit in improving insulin resistance and adipokine levels. Indicators such as fasting glucose, due to high within-subgroup statistical heterogeneity, yielded no definitive estimates from the available data. The analysis indicates that the activity status of the control group (active exercise or non-exercise) is a substantive moderating factor influencing effect size estimation, and the relevant conclusions must be interpreted cautiously at the subgroup level. Given that the overall GRADE certainty of evidence ranges from very low to moderate, the number of trials is limited, and the variability in intervention protocols is considerable, the available evidence is insufficient to support the formulation of a standardized clinical CART exercise prescription. Future research priorities should focus on adopting standardized combined aerobic and resistance training protocols, establishing consistent and clearly defined comparator conditions, and conducting randomized controlled trials with longer follow-up durations, in order to further confirm the long-term clinical benefits of CART for cardiometabolic health in adolescents.
