# Supplementary material for: Effects of combined aerobic and resistance training on cardiometabolic risk factors in overweight/obese adolescents: a systematic review and meta-analysis
Source: Front Public Health. 2026 Jul 6;14:1891252. doi: 10.3389/fpubh.2026.1891252 (PMC13381428; doi:10.3389/fpubh.2026.1891252)
Supplement: Supplementary file 1 [file Supplementary_file_1.docx]

### Supplementary Text 1 Search Strategies

#### 1. PubMed (via NCBI)

**Search Date:** May 13, 2026

**Total Results Identified:** 201

| Step | Search Terms & Boolean Logic |
| --- | --- |
| #1 | "Adolescent"[MeSH Terms] OR "Child"[MeSH Terms] |
| #2 | "child*"[Title/Abstract] OR "adolescent*"[Title/Abstract] OR "youth*"[Title/Abstract] OR "pediatric*"[Title/Abstract] OR "teenager*"[Title/Abstract] OR "student*"[Title/Abstract] |
| #3 | #1 OR #2 |
| #4 | "combined training"[Title/Abstract] OR "concurrent training"[Title/Abstract] OR "combined exercise"[Title/Abstract] OR "multimodal training"[Title/Abstract] OR "multicomponent intervention"[Title/Abstract] |
| #5 | ("aerobic"[Title/Abstract] AND "resistance"[Title/Abstract] AND ("training"[Title/Abstract] OR "exercise"[Title/Abstract])) |
| #6 | #4 OR #5 |
| #7 | "blood pressure"[MeSH Terms] OR "insulin resistance"[MeSH Terms] OR "glucose"[MeSH Terms] OR "lipids"[MeSH Terms] OR "dyslipidemias"[MeSH Terms] |
| #8 | "arterial stiffness"[Title/Abstract] OR "vascular stiffness"[Title/Abstract] OR "pulse wave velocity"[Title/Abstract] OR "PWV"[Title/Abstract] OR "brachial-ankle pulse wave velocity"[Title/Abstract] OR "carotid-femoral pulse wave velocity"[Title/Abstract] OR "endothelial function"[Title/Abstract] OR "flow-mediated dilation"[Title/Abstract] OR "FMD"[Title/Abstract] OR "intima-media thickness"[Title/Abstract] OR "cIMT"[Title/Abstract] OR "blood pressure"[Title/Abstract] OR "insulin resistance"[Title/Abstract] OR "HOMA-IR"[Title/Abstract] OR "glycemic control"[Title/Abstract] OR "cardiometabolic risk"[Title/Abstract] OR "metabolic syndrome"[Title/Abstract] OR "cardiometabolic"[Title/Abstract] |
| #9 | #7 OR #8 |
| #10 | "randomized controlled trial"[Publication Type] OR "Randomized Controlled Trial"[MeSH Terms] |
| #11 | "random*"[Title/Abstract] OR "trial"[Title/Abstract] OR "controlled"[Title/Abstract] |
| #12 | #10 OR #11 |
| #13 | #3 AND #6 AND #9 AND #12 |

### 2. Web of Science (Core Collection)

### Search Date: May 13, 2026

**Total Results Identified:** 221

| Step | Search Terms & Boolean Logic |
| --- | --- |
| #1 | TS=((adolescent* OR child* OR youth* OR pediatric* OR teenager* OR student*)) |
| #2 | TS=(("combined training" OR "concurrent training" OR "combined exercise" OR "multimodal training" OR "multicomponent intervention")) |
| #3 | TS=(("aerobic" AND "resistance" AND ("training" OR "exercise"))) |
| #4 | #2 OR #3 |
| #5 | TS=(("arterial stiffness" OR "vascular stiffness" OR "pulse wave velocity" OR "PWV" OR "endothelial function" OR "flow-mediated dilation" OR "FMD" OR "intima-media thickness" OR "cIMT" OR "blood pressure" OR "insulin resistance" OR "HOMA-IR" OR "glucose" OR "glycemic control" OR "lipids" OR "dyslipidemias" OR "cardiometabolic")) |
| #6 | TS=(random* OR trial OR "controlled study") |
| #7 | #1 AND #4 AND #5 AND #6 |

### 3. Embase (via Elsevier)

### Search Date: May 13, 2026

**Total Results Identified:** 1721

| Step | Search Terms & Boolean Logic |
| --- | --- |
| #1 | adolescent*:ab,ti OR child*:ab,ti OR youth*:ab,ti OR pediatric*:ab,ti OR teenager*:ab,ti OR student*:ab,ti |
| #2 | combined training':ab,ti OR 'concurrent training':ab,ti OR 'combined exercise':ab,ti OR 'multimodal training':ab,ti OR 'multicomponent intervention':ab,ti |
| #3 | (aerobic:ab,ti AND resistance:ab,ti AND (training:ab,ti OR exercise:ab,ti)) |
| #4 | #2 OR #3 |
| #5 | arterial stiffness':ab,ti OR 'vascular stiffness':ab,ti OR 'pulse wave velocity':ab,ti OR 'pwv':ab,ti OR 'endothelial function':ab,ti OR 'flow-mediated dilation':ab,ti OR 'fmd':ab,ti OR 'intima-media thickness':ab,ti OR 'cimt':ab,ti OR 'blood pressure':ab,ti OR 'insulin resistance':ab,ti OR 'homa-ir':ab,ti OR 'glucose':ab,ti OR 'glycemic control':ab,ti OR 'lipids':ab,ti OR 'dyslipidemias':ab,ti OR 'cardiometabolic':ab,ti |
| #6 | random*:ab,ti OR trial:ab,ti OR 'controlled study':ab,ti |
| #7 | #1 AND #4 AND #5 AND #6 |

### 4.The Cochrane Library (CENTRAL)

### Search Date: May 13, 2026

**Total Results Identified:** 1619

| Step | Search Terms & Boolean Logic |
| --- | --- |
| #1 | (adolescent* OR child* OR youth* OR pediatric* OR teenager* OR student*):ti,ab,kw |
| #2 | ("combined training" OR "concurrent training" OR "combined exercise" OR "multimodal training" OR "multicomponent intervention"):ti,ab,kw |
| #3 | (aerobic AND resistance AND (training OR exercise)):ti,ab,kw |
| #4 | #2 OR #3 |
| #5 | ("arterial stiffness" OR "vascular stiffness" OR "pulse wave velocity" OR "PWV" OR "endothelial function" OR "flow-mediated dilation" OR "FMD" OR "intima-media thickness" OR "cIMT" OR "blood pressure" OR "insulin resistance" OR "HOMA-IR" OR "glucose" OR "glycemic control" OR "lipids" OR "dyslipidemias" OR "cardiometabolic"):ti,ab,kw |
| #6 | #1 AND #4 AND #5 |

### 5. Scopus (via Elsevier)

### Search Date: May 13, 2026

**Total Results Identified:** 472

| Step | Search Terms & Boolean Logic |
| --- | --- |
| #1 | TITLE-ABS-KEY ( adolescent* OR child* OR youth* OR pediatric* OR teenager* OR student* ) |
| #2 | TITLE-ABS-KEY ( "combined training" OR "concurrent training" OR "combined exercise" OR "multimodal training" OR "multicomponent intervention" ) |
| #3 | TITLE-ABS-KEY ( aerobic AND resistance AND ( training OR exercise ) ) |
| #4 | #2 OR #3 |
| #5 | TITLE-ABS-KEY ( "arterial stiffness" OR "vascular stiffness" OR "pulse wave velocity" OR "PWV" OR "endothelial function" OR "flow-mediated dilation" OR "FMD" OR "intima-media thickness" OR "cIMT" OR "blood pressure" OR "insulin resistance" OR "HOMA-IR" OR "glucose" OR "glycemic control" OR "lipids" OR "dyslipidemias" OR "cardiometabolic" ) |
| #6 | TITLE-ABS-KEY ( random* OR trial OR "controlled study" ) |
| #7 | #1 AND #4 AND #5 AND #6 |
