# Supplementary material for: Effects of combined aerobic and resistance training on cardiometabolic risk factors in overweight/obese adolescents: a systematic review and meta-analysis
Source: Front Public Health. 2026 Jul 6;14:1891252. doi: 10.3389/fpubh.2026.1891252 (PMC13381428; doi:10.3389/fpubh.2026.1891252)
Supplement: Supplementary file 2 [file Supplementary_file_2.docx]

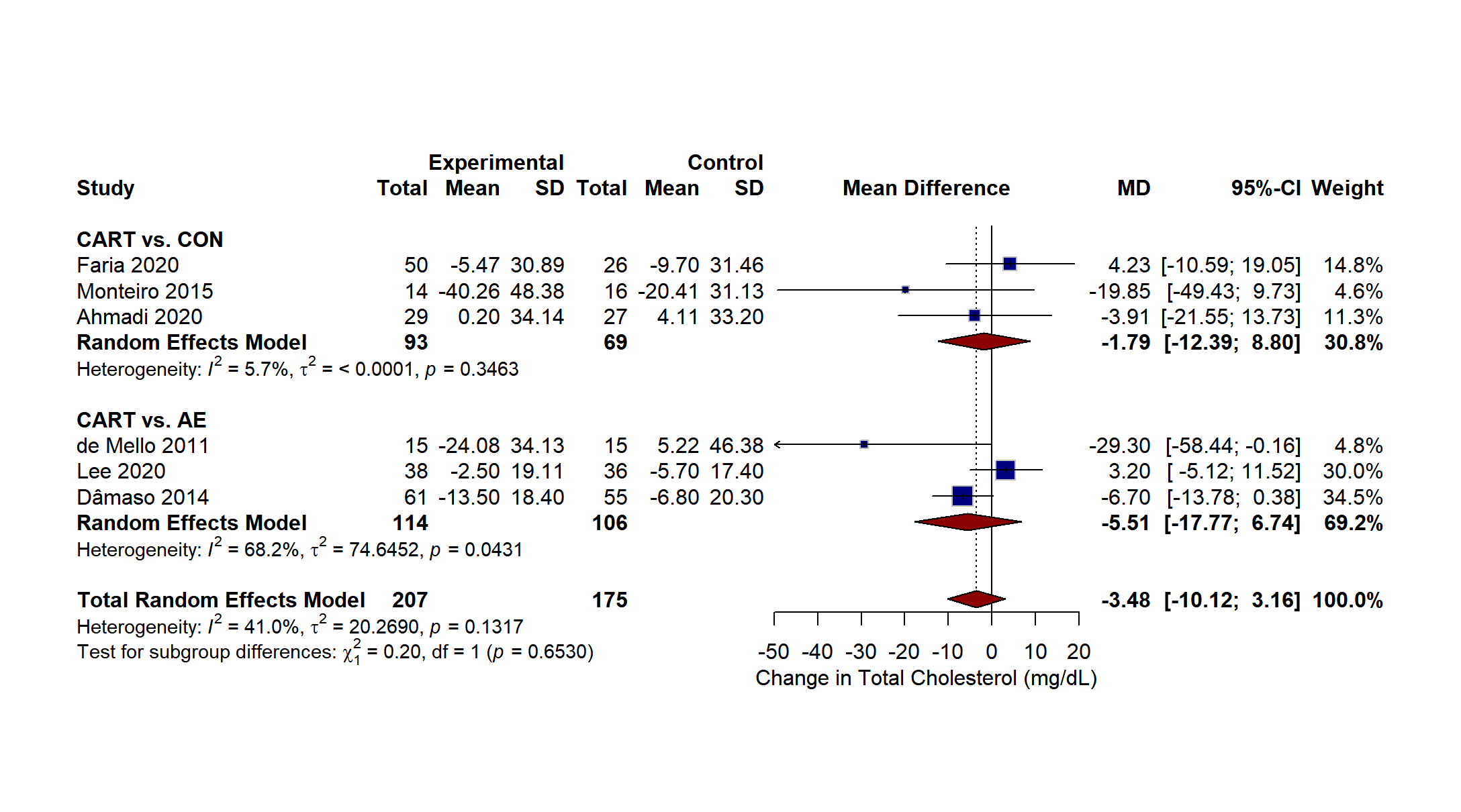


Supplementary Figure 1 Forest plot of combined exercise training versus control/aerobic exercise on total cholesterol (TC) in overweight/obese adolescents.


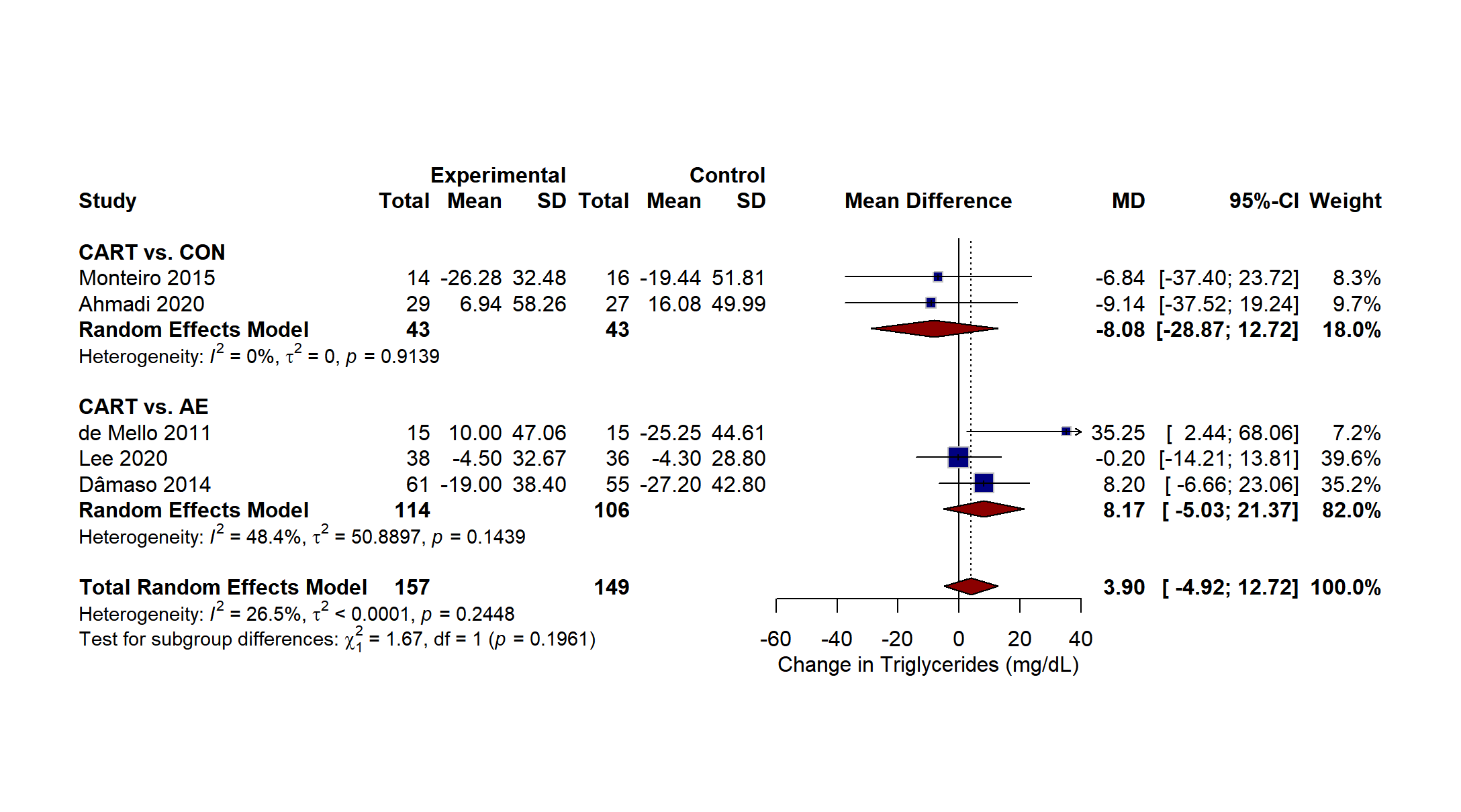


Supplementary Figure 2 Forest plot of combined exercise training versus control/aerobic exercise on triglycerides (TG) in overweight/obese adolescents.


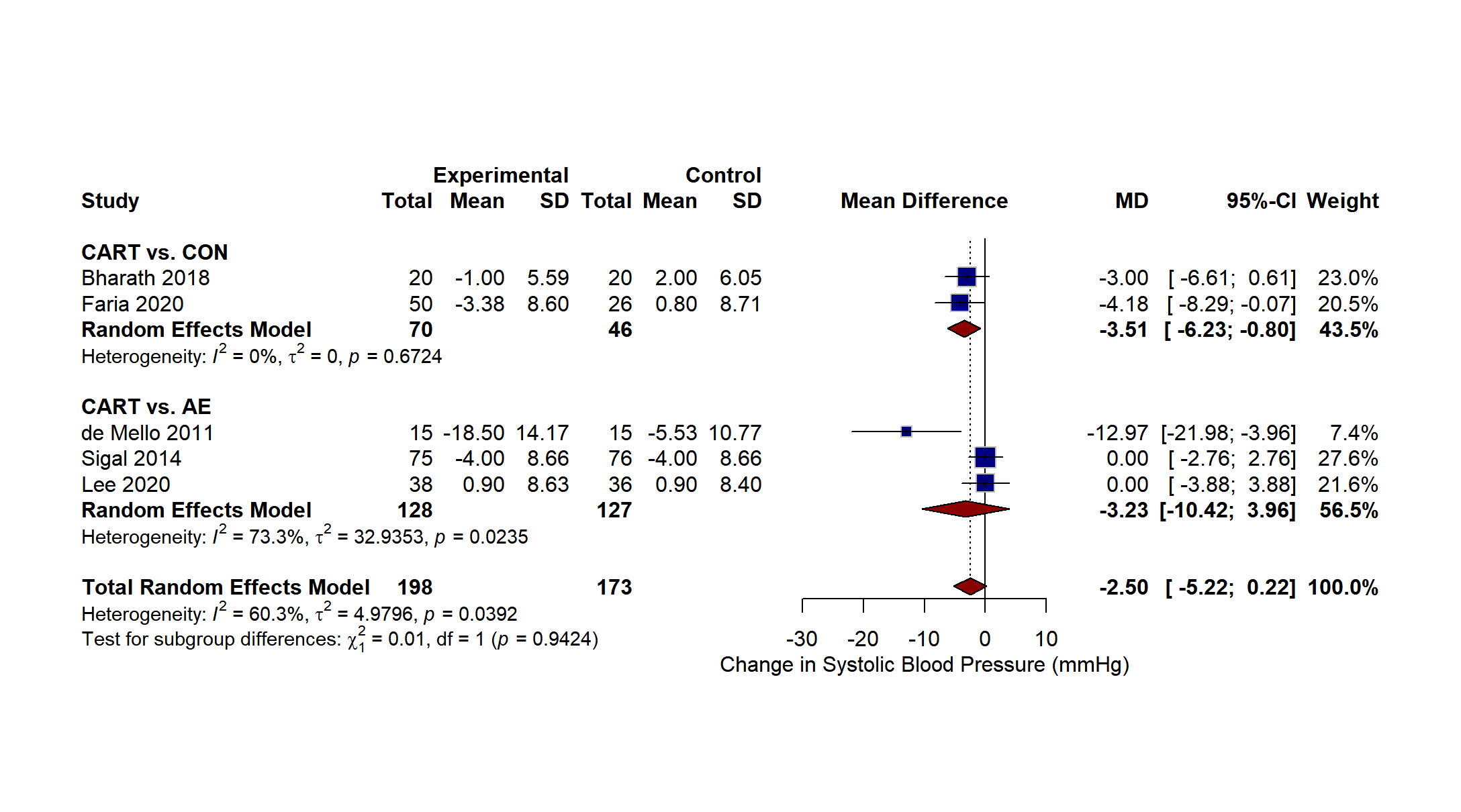


Supplementary Figure 3 Forest plot of combined exercise training versus control/aerobic exercise on systolic blood pressure (SBP) in overweight/obese adolescents.


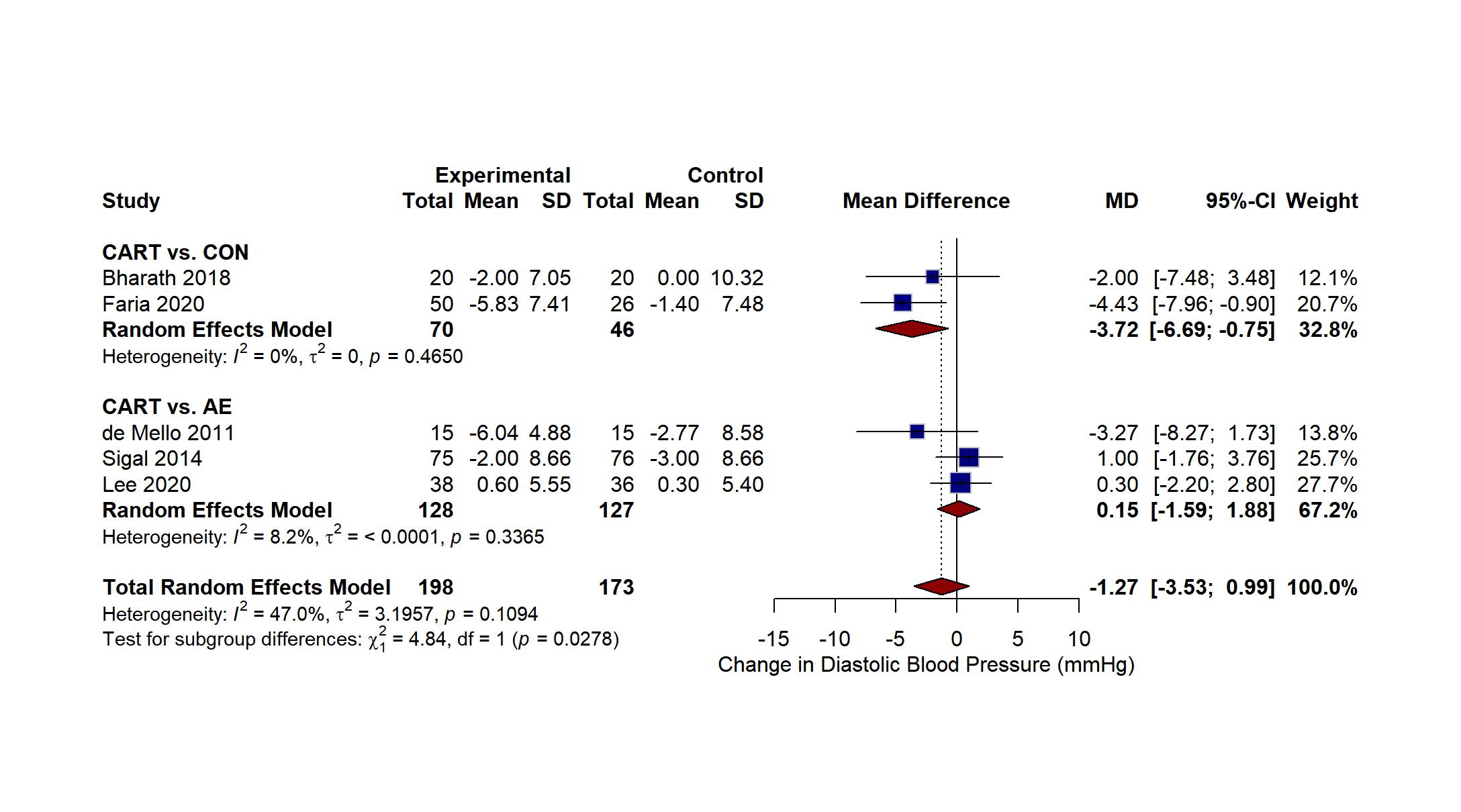


Supplementary Figure 4 Forest plot of combined exercise training versus control/aerobic exercise on diastolic blood pressure (DBP) in overweight/obese adolescents.


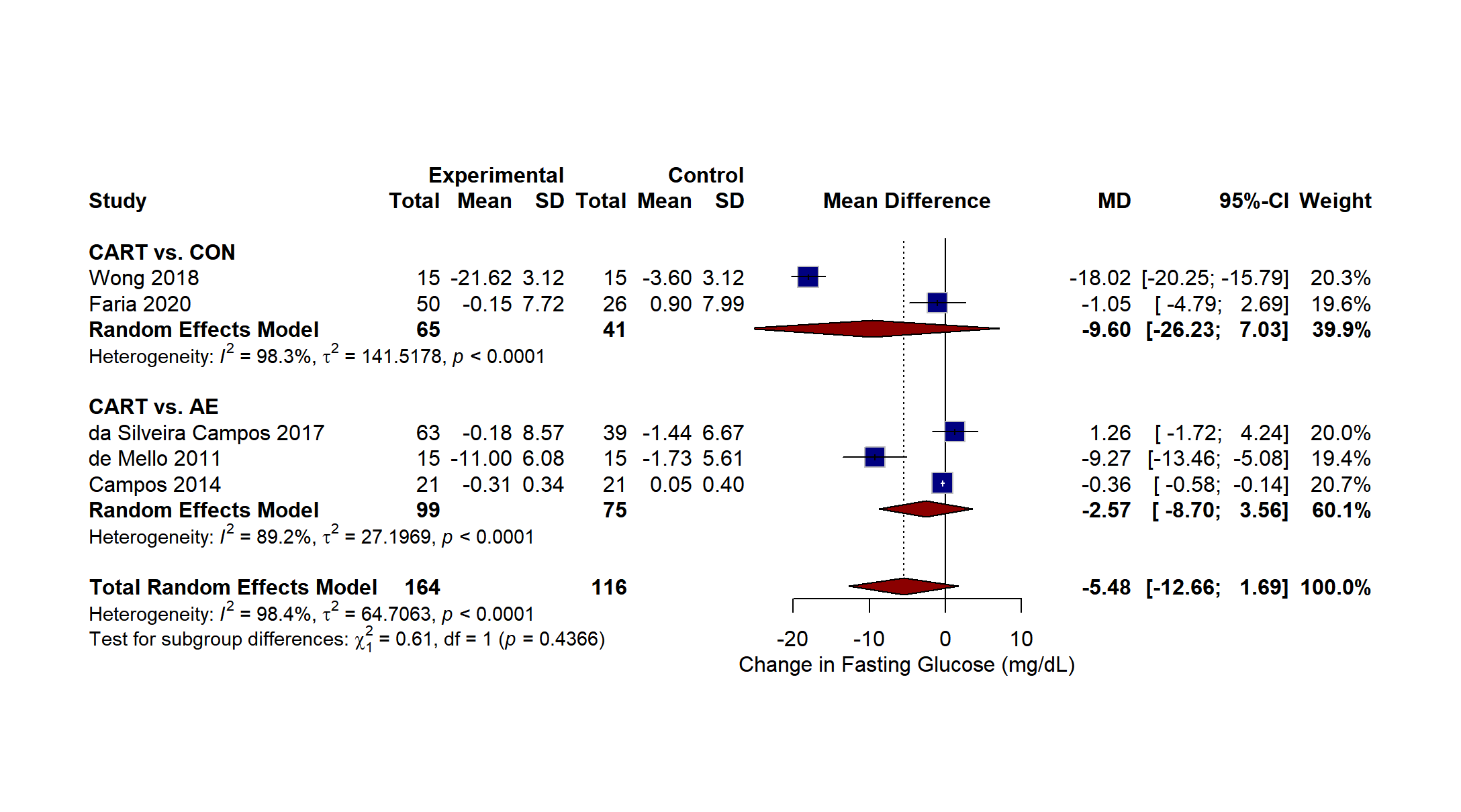


Supplementary Figure 5 Forest plot of combined exercise training versus control/aerobic exercise on fasting glucose in overweight/obese adolescents.


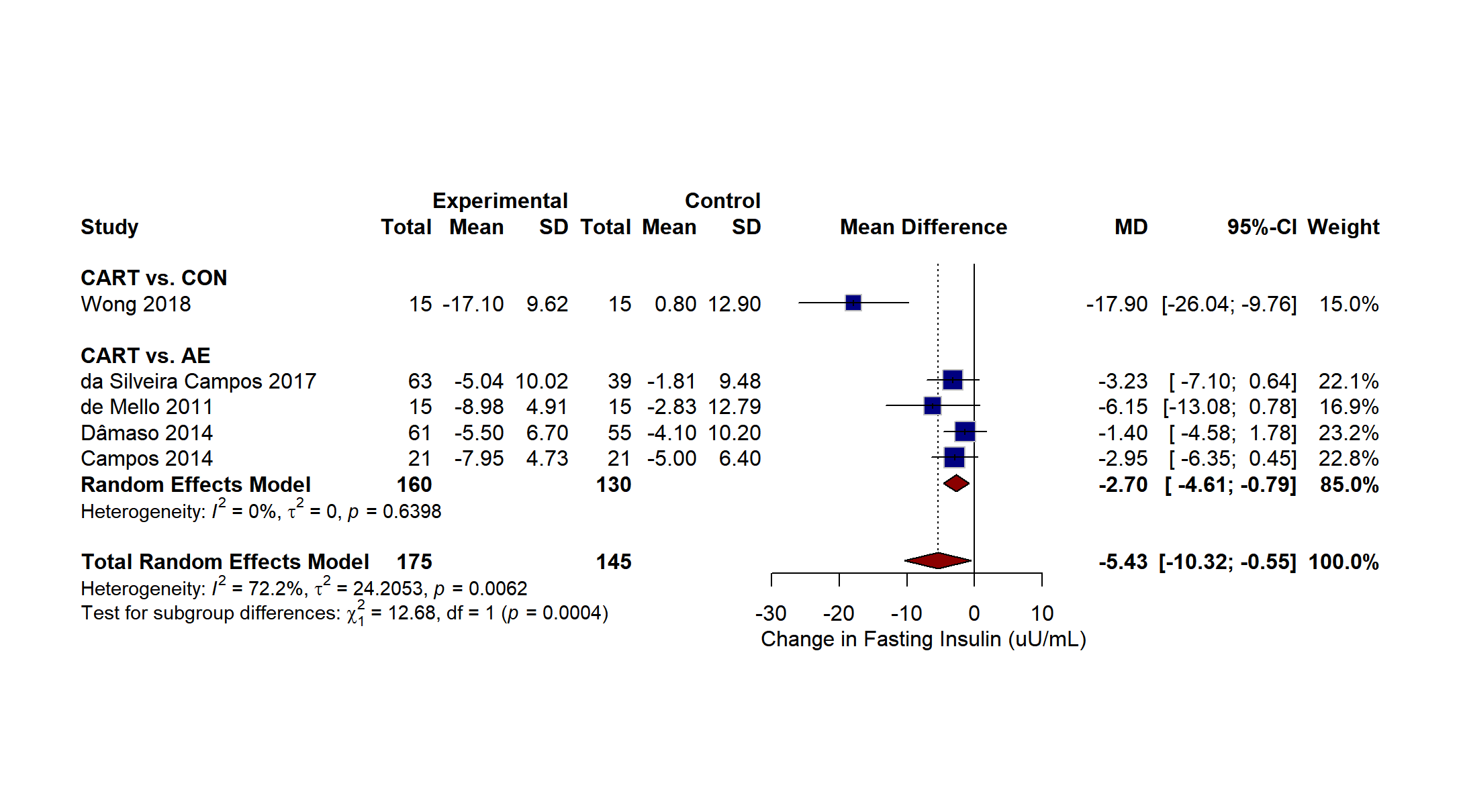


Supplementary Figure 6 Forest plot of combined exercise training versus control/aerobic exercise on fasting insulin in overweight/obese adolescents.


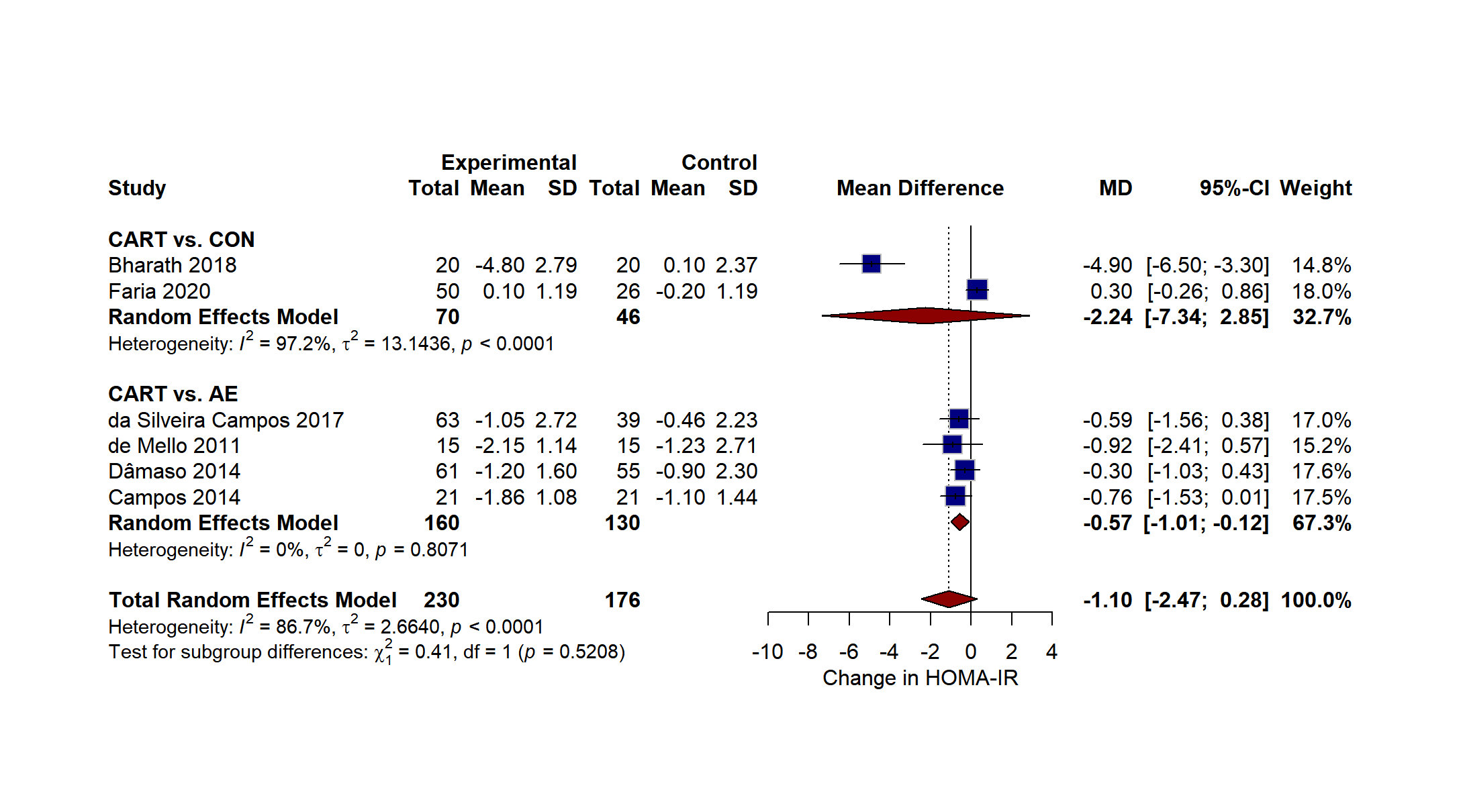


Supplementary Figure 7 Forest plot of combined exercise training versus control/aerobic exercise on HOMA-IR in overweight/obese adolescents.


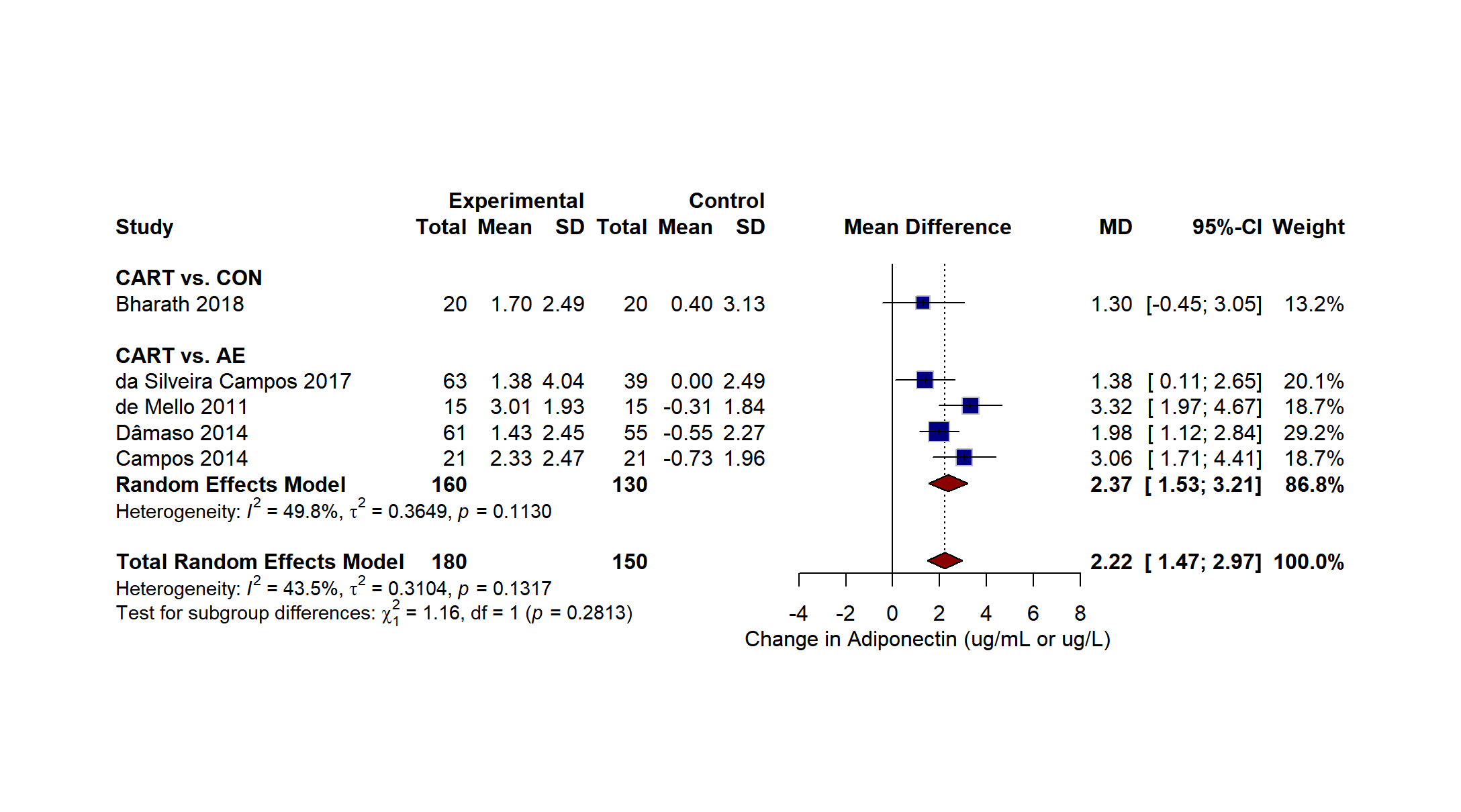


Supplementary Figure 8 Forest plot of combined exercise training versus control/aerobic exercise on adiponectin in overweight/obese adolescents.
